# Leucine-973 is a crucial residue differentiating insulin and IGF-1 receptor signaling

**DOI:** 10.1172/JCI161472

**Published:** 2023-02-15

**Authors:** Hirofumi Nagao, Weikang Cai, Bruna B. Brandão, Nicolai J. Wewer Albrechtsen, Martin Steger, Arijeet K. Gattu, Hui Pan, Jonathan M. Dreyfuss, F. Thomas Wunderlich, Matthias Mann, C. Ronald Kahn

**Affiliations:** 1 Section of Integrative Physiology and Metabolism, Joslin Diabetes Center, Harvard Medical School, Boston, Massachusetts, USA.; 2 Department of Biomedical Sciences, New York Institute of Technology College of Osteopathic Medicine, Old Westbury, New York, USA.; 3 Department of Proteomics and Signal Transduction, Max Planck Institute of Biochemistry, Martinsried, Germany.; 4 Novo Nordisk Foundation Center for Protein Research, Faculty of Health and Medical Sciences,and; 5 Department of Clinical Biochemistry, Rigshospitalet, University of Copenhagen, Copenhagen, Denmark.; 6 Division of Endocrinology, Diabetes and Hypertension, Brigham and Women’s Hospital, Boston, Massachusetts, USA.; 7 Bioinformatics and Biostatistics Core, Joslin Diabetes Center, Harvard Medical School, Boston, Massachusetts, USA.; 8 Max Planck Institute for Metabolism Research, Center for Endocrinology, Diabetes and Preventive Medicine, University Hospital of Cologne, Center for Molecular Medicine Cologne, and; 9 Excellence Cluster on Cellular Stress Responses in Aging Associated Diseases, University of Cologne, Cologne, Germany.

**Keywords:** Endocrinology, Metabolism, Insulin signaling

## Abstract

Insulin and IGF-1 receptors (IR and IGF1R) are highly homologous and share similar signaling systems, but each has a unique physiological role, with IR primarily regulating metabolic homeostasis and IGF1R regulating mitogenic control and growth. Here, we show that replacement of a single amino acid at position 973, just distal to the NPEY motif in the intracellular juxtamembrane region, from leucine, which is highly conserved in IRs, to phenylalanine, the highly conserved homologous residue in IGF1Rs, resulted in decreased IRS-1/PI3K/Akt/mTORC1 signaling and increased Shc/Gab1/MAPK cell cycle signaling. As a result, cells expressing L973F-IR exhibited decreased insulin-induced glucose uptake, increased cell growth, and impaired receptor internalization. Mice with knockin of the L973F-IR showed similar alterations in signaling in vivo, and this led to decreased insulin sensitivity, a modest increase in growth, and decreased weight gain when mice were challenged with a high-fat diet. Thus, leucine-973 in the juxtamembrane region of the IR acts as a crucial residue differentiating IR signaling from IGF1R signaling.

## Graphical abstract

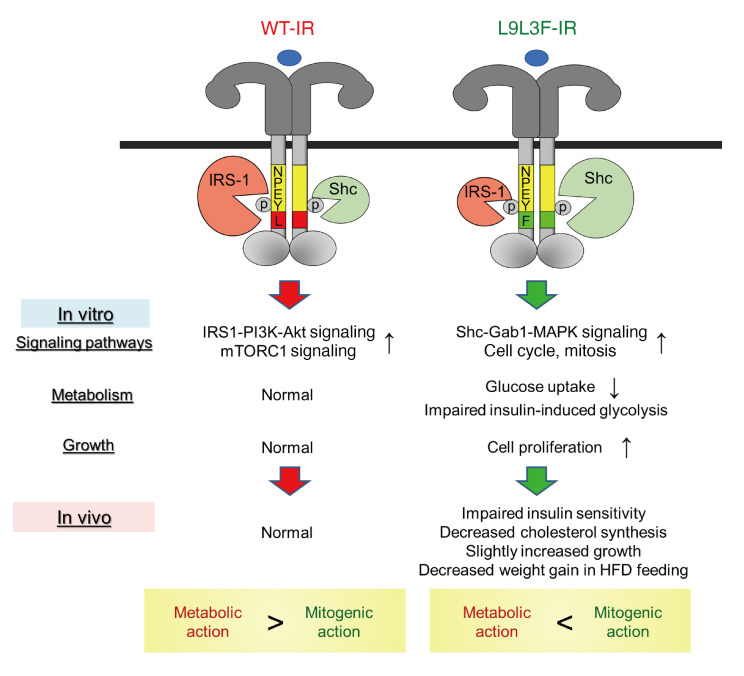

## Introduction

Insulin and IGF-1 mediate their pleiotropic biological effects by binding to insulin and IGF-1 receptors (IR and IGF1R) on the surface of the cell (1–3). Due to the high degree of homology between IR and IGF1R, these receptors share many overlapping downstream signaling pathways (1). Activation of IR and IGF1R by their cognate ligands initiates a cascade of phosphorylation events beginning with a conformational change of the receptors leading to autophosphorylation and the recruitment and phosphorylation of substrates such as IRS-1 and Shc proteins (4–6). This results in activation of two major canonical signaling pathways: the IRS-1/PI3K/Akt pathway, which is linked to most metabolic actions, such as stimulation of glucose uptake, lipogenesis, glycogen synthesis, and inhibition of gluconeogenesis, and the Shc/Grb2/Sos/Ras/Raf/MAPK pathway, which regulates cell growth and differentiation (5, 7). Despite their similarity in signaling, there are major differences between the actions of insulin and IGF-1 in vivo. Insulin is the dominant metabolic hormone controlling glucose and lipid homeostasis, such that humans or mice with insulin deficiency or mutations of the IR develop severe hyperglycemia and uncontrolled lipolysis (8–10), whereas animals lacking IGF-1 or with mutations in IGF1R display severe growth deficiency both prenatally (11) and postnatally (12, 13).

Recently, using chimeric receptors, we have demonstrated how domain differences between IR and IGF1R contribute to the distinct functions of these receptors (14). Thus, receptors with the intracellular domain of IGF1R showed increased activation of Shc and Gab-1 and more potent regulation of genes involved in proliferation, whereas those with the intracellular domain of IR showed higher IRS-1 phosphorylation and stronger regulation of genes involved in metabolic pathways (14). Moreover, using global phosphoproteomics, we showed that IR preferentially stimulates phosphorylation on proteins associated with the Akt and mechanistic target of rapamycin complex 1 (mTORC1) pathways, whereas IGF1R preferentially stimulates phosphorylation of proteins associated with the Rho family of GTPases and cell cycle progression (15). The juxtamembrane regions of these receptors, especially the NPEY motifs, have been shown to be critical for recruiting receptor substrates, including IRS-1 and Shc (16, 17). Indeed, tyrosine-972 in the NPEY motif of IR (numbering relative to human IR-B or +exon 11 isoform), which is phosphorylated following ligand stimulation, serves as a docking site for proteins with a PTB domain, such as IRS-1 and Shc, and mutation of this residue markedly decreases phosphorylation of these receptor substrates and activation of downstream signaling (16, 17). Likewise, humans with a naturally occurring mutation of proline-970 in the NPEY motif of the IR to threonine exhibit severe insulin resistance (18). Other receptor tyrosine kinases (RTKs), such as the epidermal growth factor (EGF) receptor, and some nonkinase receptors, such as the LDL receptor (LDL-R), have similar NPXY motifs (19), although in the latter, this site is not tyrosine phosphorylated. However, in the LDL-R as well as in the IR and IGF1R, this NPXY motif has a role in internalization of the receptors (20–22). Thus, after ligand binding, the insulin-IR complex is internalized, followed by degradation of the ligand and either degradation, recycling, or translocation of the receptor to other intracellular sites (23–25). Disturbance in IR internalization leads to hyperinsulinemia by prolonging insulin half-life in the circulation (23), a process that may also be disrupted in insulin-resistant obese individuals and patients with type 2 diabetes mellitus (23, 24).

By sequential substitution of all amino acids that differ in the juxtamembrane region of IR versus IGF1R, we have previously shown that another important sequence difference in the region surrounding the NPEY motif of IR and IGF1R may be located at position 973 just C-terminal to motif. This residue is a leucine in IR and a phenylalanine in IGF1R, and we have found that the replacement of leucine-973 in the juxtamembrane region of IR with Phe decreases IRS-1 binding and increases Shc binding. In the present study, we have explored in depth this important region of these receptors both in vitro and in vivo by generating cell lines in which we have deleted both endogenous IR and IGF1R and then reconstituted the cells with either WT human IR B isoform (WT-IR) or a mutant IR in which leucine-973 has been changed to phenylalanine (L973F-IR) as well as by creation of a knockin mouse model with an *INSR* L973F substitution. Here, we show that substitution of phenylalanine for leucine at position 973 in the IR decreases IRS-1/PI3K/Akt/mTORC1 signaling, upregulates Shc/Gab1/MAPK signaling and cell cycle–related pathways, and decreases ligand-stimulated receptor internalization in vitro. Functionally, this leads to decreased insulin-induced glycolysis and an increased proliferation rate at the cellular level. Mice with knockin of the L973F-IR show similar signaling alterations, which lead to impaired insulin sensitivity, increases in growth, and resistance to weight gain upon a high-fat diet (HFD) challenge. Thus, leucine-973 in the +1 position following the NPEY motif in the juxtamembrane region is a key residue differentiating IR signaling from IGF1R signaling.

## Results

### L973F-IR mutation mimics IGF1R signaling.

**Figure 1:**
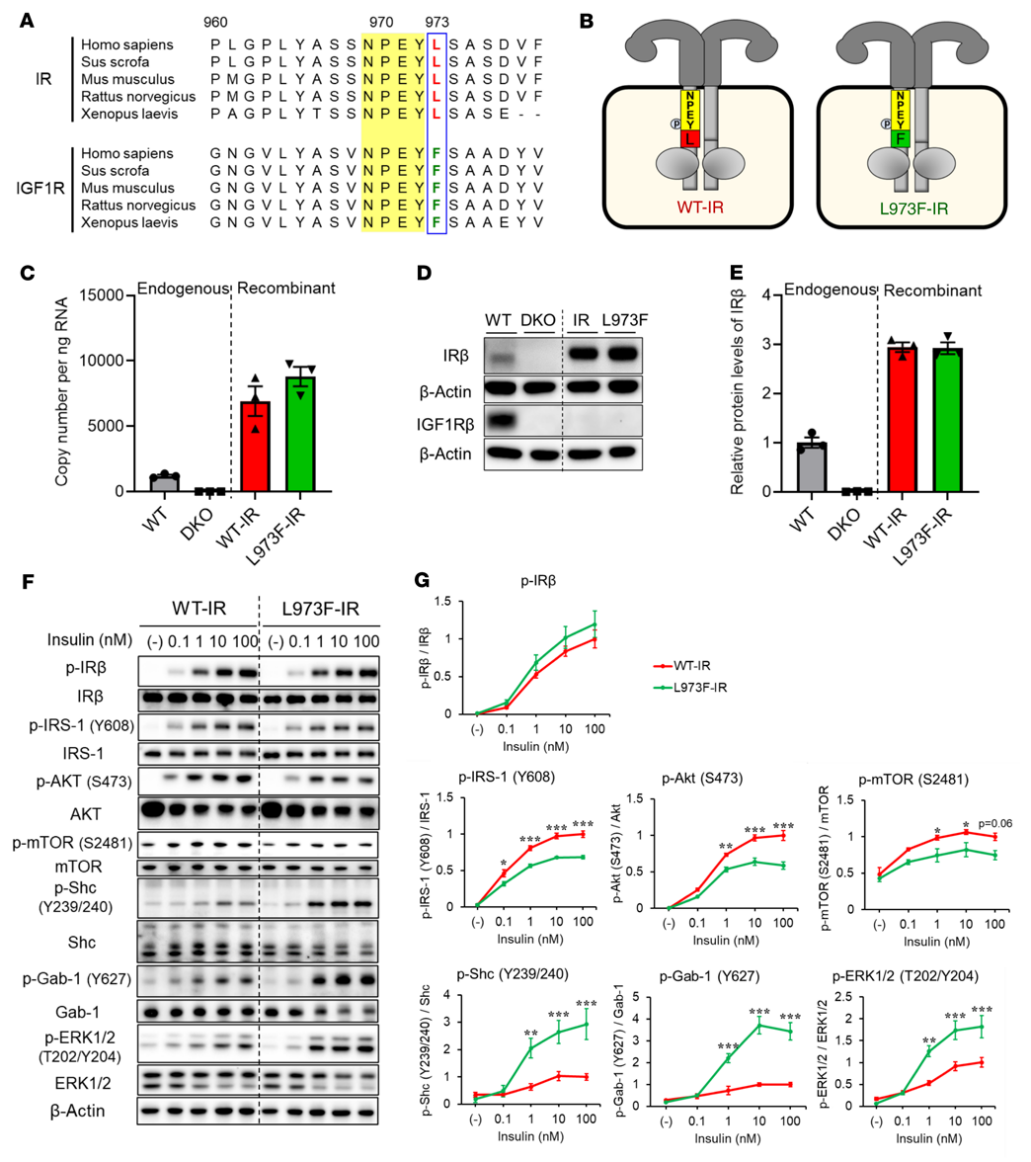
Generation and characterization of the L973F-IR in vitro. (**A**) Aligned sequences surrounding the NPEY motif in the juxtamembrane regions of the insulin and IGF1Rs in several species are shown, illustrating the constant leucine in the position in IR and phenylalanine in IGF1R. (**B**) Schematic representation of IR/IGF1R DKO preadipocytes reconstituted with WT-IR and L973F mutated IR (L973F-IR). (**C**) Relative mRNA levels of recombinant receptors of WT-IR and L973F-IR as determined by qPCR using cDNA standards for quantitation. Data are represented as mean ± SEM copy number per ng total RNA (*n* = 3). (**D**) Immunoblotting of IR and IGF1R using antibodies specific for IRβ (sc-711, Santa Cruz Biotechnology Inc.) and IGF1Rβ in lysates from WT, DKO, WT-IR, and L973F-IR preadipocytes. (**E**) Densitometric analysis of IRβ (sc-711, Santa Cruz Biotechnology Inc.) by immunoblotting. The level of IRβ in WT cells was set at 1. Data are represented as mean ± SEM (*n* = 3). (**F**) Immunoblotting of the phosphorylation of IR, IRS-1, Akt, mTOR, Shc, Gab-1, and ERK in lysates from preadipocytes expressing WT-IR and L973F-IR stimulated with 0, 0.1, 1, 10, or 100 nM insulin for 15 minutes. (**G**) Densitometric analysis of p-IR, p–IRS-1, p-Akt, p-mTOR, p-Shc, p–Gab-1, and p-ERK following insulin stimulation. The level of each phosphorylated protein in WT-IR cells stimulated with 100 nM insulin was set at 1. Data are represented as mean ± SEM (*n* = 4). *P < 0.05; **P < 0.01; ***P < 0.001, WT-IR versus L973F-IR, 2-way ANOVA.

The NPEY motif in the juxtamembrane region in both IR and IGF1R is critical for recruitment of receptor substrates including IRS-1 and Shc (16, 17). We have previously shown that, in the region surrounding the NPEY motif, 4 out of 16 residues differ between the IR and IGF1R (equivalent to Pro963, Ser968, Leu973, and Ser976 in the IR) and by systematic substitution of each of these residues showed that only replacement of leucine-973, the residue in the +1 position following the NPEY motif, with phenylalanine results in a shift from binding IRS-1 to binding Shc in a receptor pull-down assay (14). Consistent with its importance, the presence of leucine following the NPEY motif in IR versus phenylalanine in the IGF1R is conserved among different species (Figure 1A). To analyze the effects on replacement of leucine-973 in the juxtamembrane region with phenylalanine, we generated preadipocytes in which both endogenous IR and IGF1R have been genetically inactivated to create double-knockout (DKO) cells using a Cre-lox recombination and then reconstituted these DKO cells with WT-IR or L973F-IR, both of which were FLAG tagged (Figure 1B).

As expected, IR and IGFR were not detected in DKO cells, while cells reconstituted with WT-IR or L973F-IR had similar expression of these receptors, as judged by mRNA (Figure 1C) and protein levels (Figure 1, D and E). Using an IRβ antibody specifically recognizing epitopes surrounding tyrosine-972 of the IR (CST3025, Cell Signaling Technology), we found high reactivity to the WT-IR, whereas the same antibody showed a dramatic reduction of reactivity toward the L973F-IR (Supplemental Figure 1, A and B; supplemental material available online with this article; https://doi.org/10.1172/JCI161472DS1). To determine potential differences between WT-IR and L973F-IR in insulin signaling, we stimulated WT-IR and L973F-IR preadipocytes with 0, 0.1, 1, 10, and 100 nM insulin for 15 minutes and assessed downstream signaling. While insulin stimulation induced robust tyrosine autophosphorylation of both receptors with dose response to insulin (Figure 1, F and G), the degree of activation of the downstream signaling cascades differed. Thus, insulin induced much more robust phosphorylation of IRS-1^Y608^, Akt^S473^, and mTOR^S2481^ in cells with WT-IR than in cells expressing L973F-IR (Figure 1G). Thus, L973F-IR cells showed decreased responsiveness to insulin stimulation in phosphorylation of IRS-1^Y608^, Akt^S473^, and mTOR^S2481^ at all concentrations, from 0.1 to 100 nM. Phosphorylation of Foxo1 and PRAS40, direct downstream targets of Akt, also showed decreased response to insulin in L973F-IR cells (Supplemental Figure 1, C and D). In contrast, L973F-IR cells displayed 2- to 4-fold increases in Shc, Gab-1, and ERK phosphorylation (Figure 1G) following stimulation with 1 to 100 nM insulin, consistent with an increase in Shc recruitment to the receptors compared with WT-IR cells (Supplemental Figure 1E). Together, these data show that replacing the natural leucine at position 973 of the IR with the corresponding phenylalanine found in the IGF1R alters downstream receptor signaling, favoring a mitogenic pattern compared with a metabolic pattern (Figure 1G and Supplemental Figure 1F).

### Regulation of cellular metabolism and growth by normal and L973F-IRs.

**Figure 2:**
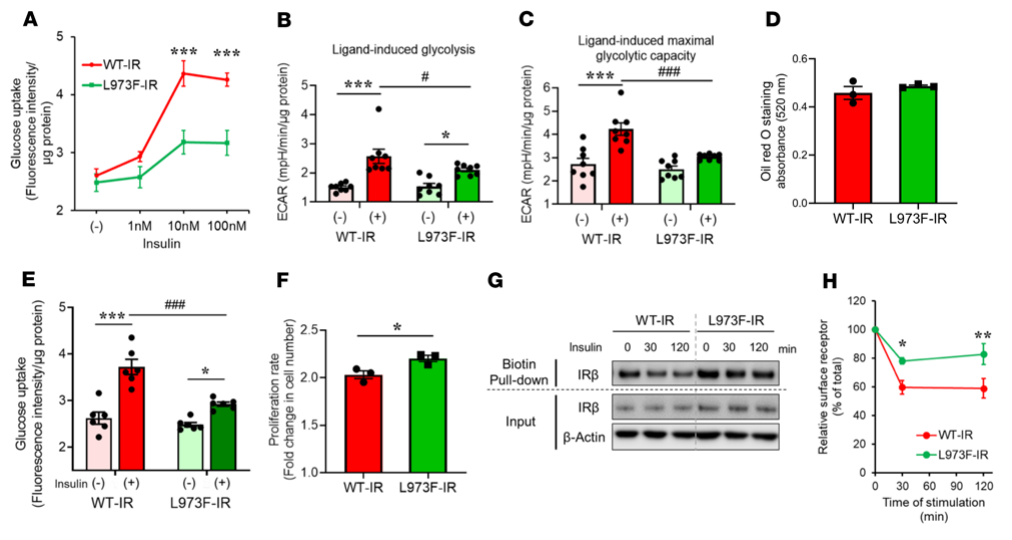
L973F-IR mutation in regulation of metabolism and growth. (**A**) Glucose uptake in WT-IR and L973F-IR preadipocytes stimulated with 0, 1, 10, or 100 nM insulin. Data are represented as mean ± SEM (*n* = 6). ***P < 0.001, WT-IR versus L973F-IR, 2-way ANOVA. (**B**) Glycolysis induced by 100 nM insulin for 6 hours. WT-IR and L973F-IR preadipocytes were FBS starved overnight before insulin stimulation. ECAR values were measured using a Seahorse X96 Bioanalyzer. Fold change of glycolysis rates in response to insulin stimulation were calculated by comparing ECARs on insulin stimulation after glucose injection. Data are represented as mean ± SEM (*n* = 8). *P < 0.05; ***P < 0.001, basal versus insulin. ^#^*P* < 0.05, WT-IR versus L973F-IR, 2-way ANOVA. (**C**) Glycolytic rate (maximal glycolytic capacity) induced by 100 nM insulin for 6 hours. Fold change of the maximal glycolytic capacity in response to insulin stimulation as measured by ECAR on insulin stimulation after oligomycin injection was calculated. Data are represented as mean ± SEM (*n* = 8). ***P < 0.001, basal versus insulin. ^###^*P* < 0.001, WT-IR versus L973F-IR, 2-way ANOVA. (**D**) Triglyceride accumulation in WT-IR and L973F-IR adipocytes assessed by oil red O staining on day 7 after induction of differentiation (*n* = 3 per group). Data are represented as mean ± SEM. (**E**) Glucose uptake in WT-IR and L973F-IR differentiated adipocytes stimulated with 10 nM insulin. Data are represented as mean ± SEM (*n* = 6). *P < 0.05; ***P < 0.001, basal versus insulin. ^###^*P* < 0.001, WT-IR versus L973F-IR, 2-way ANOVA. (**F**) Proliferation rates of WT-IR and L973F-IR preadipocytes (*n* = 3) per day are shown as mean ± SEM. *P < 0.05, unpaired Student’s *t* test. (**G**) Immunoblotting of IRβ in whole cell lysates and biotin-labeled cell surface fraction at 0, 30, and 120 minutes after 100 nM insulin stimulation. Biotinylated receptors were pulled down by streptavidin agarose. (**H**) Quantification of relative surface receptors at 0, 30, and 120 minutes after 100 nM insulin stimulation. Data are represented as mean ± SEM. *P < 0.05; **P < 0.001, WT-IR versus L973F-IR, 2-way ANOVA.

Corresponding to their differences in signaling activity, glucose uptake in L973F-IR preadipocytes showed significantly decreased responsiveness to insulin stimulation compared with WT-IR preadipocytes (Figure 2A). Metabolic activity of cells expressing the WT and L973F-IR was also assessed using a Seahorse Metabolic Bioanalyzer in the absence or presence of 100 nM insulin stimulation. Insulin induced an extracellular acidification rate in WT-IR–expressing preadipocytes, indicating increased glycolysis (Figure 2B) and maximal glycolytic capacity (Figure 2C). In contrast, L973F-IR preadipocytes showed impairment in insulin-induced glycolysis (Figure 2B) and maximal glycolytic capacity (Figure 2C), consistent with their differences in glucose uptake and signaling activity. Following exposure to a differentiation cocktail containing indomethacin, dexamethasone, IBMX, insulin, and T3, both WT-IR– and L973F-IR–expressing preadipocytes showed similar adipocyte differentiation, as measured by lipid droplet accumulation in oil red O staining (Figure 2D and Supplemental Figure 1G). When normalized to protein content, the lipid droplet accumulation in WT-IR cells was slightly higher than that in L973F-IR cells (Supplemental Figure 1H). Insulin-induced glucose uptake in L973F-IR–differentiated adipocytes was also significantly decreased compared with that in WT-IR–differentiated adipocytes (Figure 2E). On the other hand, in normal culture media, preadipocytes expressing L973F-IR showed a higher rate of cell proliferation compared with preadipocytes expressing WT-IR (Figure 2F), consistent with enhanced growth signaling by L973F-IR.

The NPEY motif in the juxtamembrane domain of a variety of membrane receptors has been shown to play a role in ligand-stimulated internalization (20–22), and this effect is more pronounced in IR than IGF1R (14). Using cell-surface biotinylation followed by streptavidin pulldown, we found that insulin stimulated rapid receptor internalization of WT-IR, with a 40% reduction of the surface-labeled receptors by 30 minutes (Figure 2, G and H). Consistent with more IGF1R-like behavior, there was reduced internalization of the L973F-IR, with only 20% internalized by 30 minutes, and this difference persisted through 120 minutes after insulin stimulation. Thus, the presence of leucine versus phenylalanine at position 973 in the juxtamembrane region of the IR plays an important role in regulating ligand-induced glycolysis, cell proliferation, and receptor internalization, with the presence of phenylalanine making the IR more IGF1R-like in behavior.

### Differential phosphoproteomic signature between WT-IR and L973F-IR cells.

**Figure 3:**
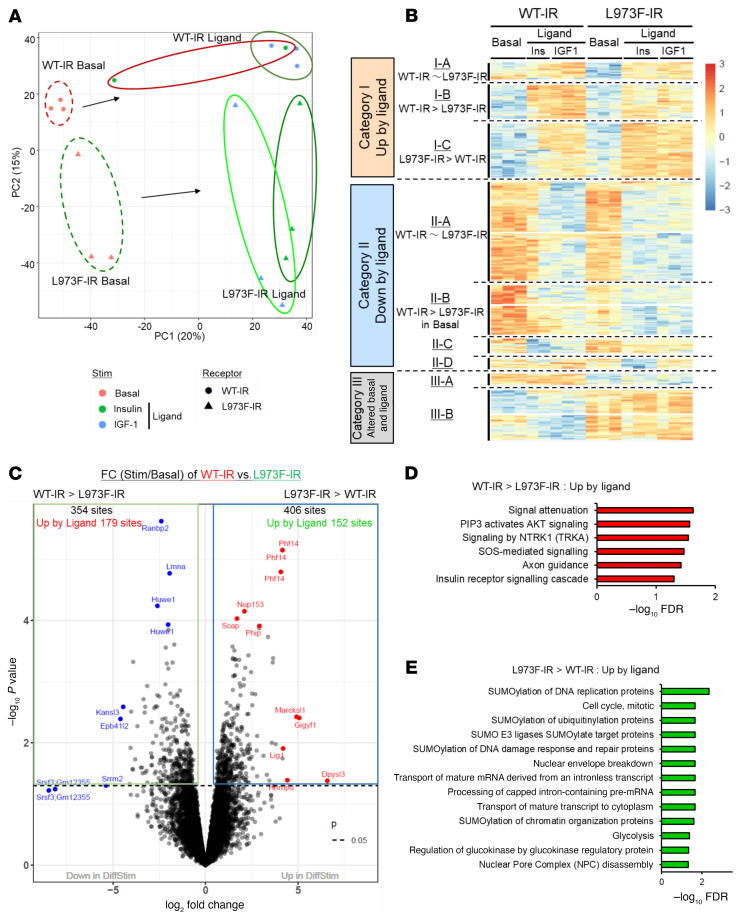
Phosphoproteomic signature of WT-IR and L973F-IR cells. (**A**) PCA of the phosphosites identified by LC-MS/MS from DKO preadipocytes reconstituted with WT-IR and L973F-IR in the basal and ligand-stimulated (100 nM insulin or IGF-1 for 15 minutes) states. Cells were serum starved for 6 hours in DMEM containing 0.1% BSA before insulin and IGF-1 stimulation. (**B**) Heatmap showing hierarchical clustering of the differential phosphopeptides in WT-IR and L973F-IR–expressing cells in the basal and ligand-stimulated states. Values are shown as *z* scores of log_2_ transformed intensity values. (**C**) Volcano plot of phosphosites between WT-IR cells and L973F-IR cells using fold changes after ligand stimulation. Changes in phosphorylation between WT-IR cells and L973F-IR cells as fold stimulation, i.e., the ligand-stimulated level divided by the basal level of the phosphosites. Differences were considered significant at *P* < 0.05. (**D**) Reactome pathway enrichment analysis of phosphosites upregulated by ligand for fold-change WT-IR > L973F-IR. Plots are –log_10_ transforms of enrichment FDR value. (**E**) Reactome pathway enrichment analysis of phosphosites upregulated by ligand for fold change L973F-IR > WT-IR. Plots are –log_10_ transformed of enrichment FDR value.

To identify the differences in the regulation of signaling networks between WT-IR and L973F-IR, we performed global phosphoproteomics (26) on WT-IR and L973F-IR cells with or without 100 nM insulin stimulation for 15 minutes. To determine whether the nature of the ligand might also influence insulin versus IGF-1–like signaling, we also treated these cells with 100 nM IGF-1 for 15 minutes. Across all conditions, an average of approximately 12,700 phosphosites were identified and quantified. Principal component analysis (PCA) displayed a clear separation of the phosphoproteome between IR and L973F-IR cells both in the basal (nonstimulated) state and after ligand stimulation (Figure 3A). Heatmap analysis with hierarchical clustering indicated the specific phosphorylation pattern between WT-IR and L973F-IR cells (Figure 3B). A total of 979 phosphosites were differentially regulated at an FDR of less than 0.05, and these could be divided into 6 groups based on phosphorylation pattern.

Of the 846 ligand-regulated phosphosites (categories I and II), 49 sites were equally upregulated (category I-A) and 288 sites were equally downregulated (category II-A) in both WT-IR and L973F-IR cells in response to ligand stimulation (up- or downregulation of these sites was not significantly changed between these 2 cell lines). At the single concentration tested (100 nM), most phosphosites were regulated almost equally by both insulin and IGF-1. For example, SRSF4^S457^ and DDX24^S80^, which are associated with mRNA splicing and alteration of RNA secondary structure, were equally upregulated by ligand in cells with either receptor (Supplemental Figure 2A), while ROCK1^S1336^ and RANBP2^S796^, which are associated with cytoskeleton remodeling and cell cycle, were equally downregulated by ligand (Supplemental Figure 2B). These overlapping signaling events between WT-IR and L973F-IR are illustrated in an integrated signaling map shown in Supplemental Figure 2C. This map includes 16 sites that were upregulated and 19 sites that were downregulated by insulin/IGF-1 stimulation. These involved several pathways known to be associated with IR/IGF1R signaling, including the PI3K/Akt pathway, mTORC1 pathways, MAPK signaling, Rho GTPase signaling, cell cycle, and mitosis. In addition, many other phosphosites were downregulated following ligand stimulation (category II, B to D), but with various patterns, depending on differences in basal and stimulated levels of phosphorylation. These included a cluster of phosphosites in which the basal phosphorylation was higher in WT-IR than in L973F-IR (II-B, 141 sites), a cluster in which ligand-dependent reduction of phosphorylation was greater in WT-IR than in L973F-IR (II-C, 41 sites), and a cluster in which phosphorylation of proteins was downregulated more by ligands in WT-IR than in L973F-IR (II-D, 28 sites). Only one small cluster (category II-D), such as SUDS3^S32^ and CPD^T1365^, was unequally regulated between insulin and IGF-1 stimulation (Supplemental Figure 2, D and E), suggesting that for some actions, there may be differential effects based on whether insulin or IGF-1 occupies the receptor.

### WT-IR– and L973F-IR–specific signaling in the phosphoproteome.

**Figure 4:**
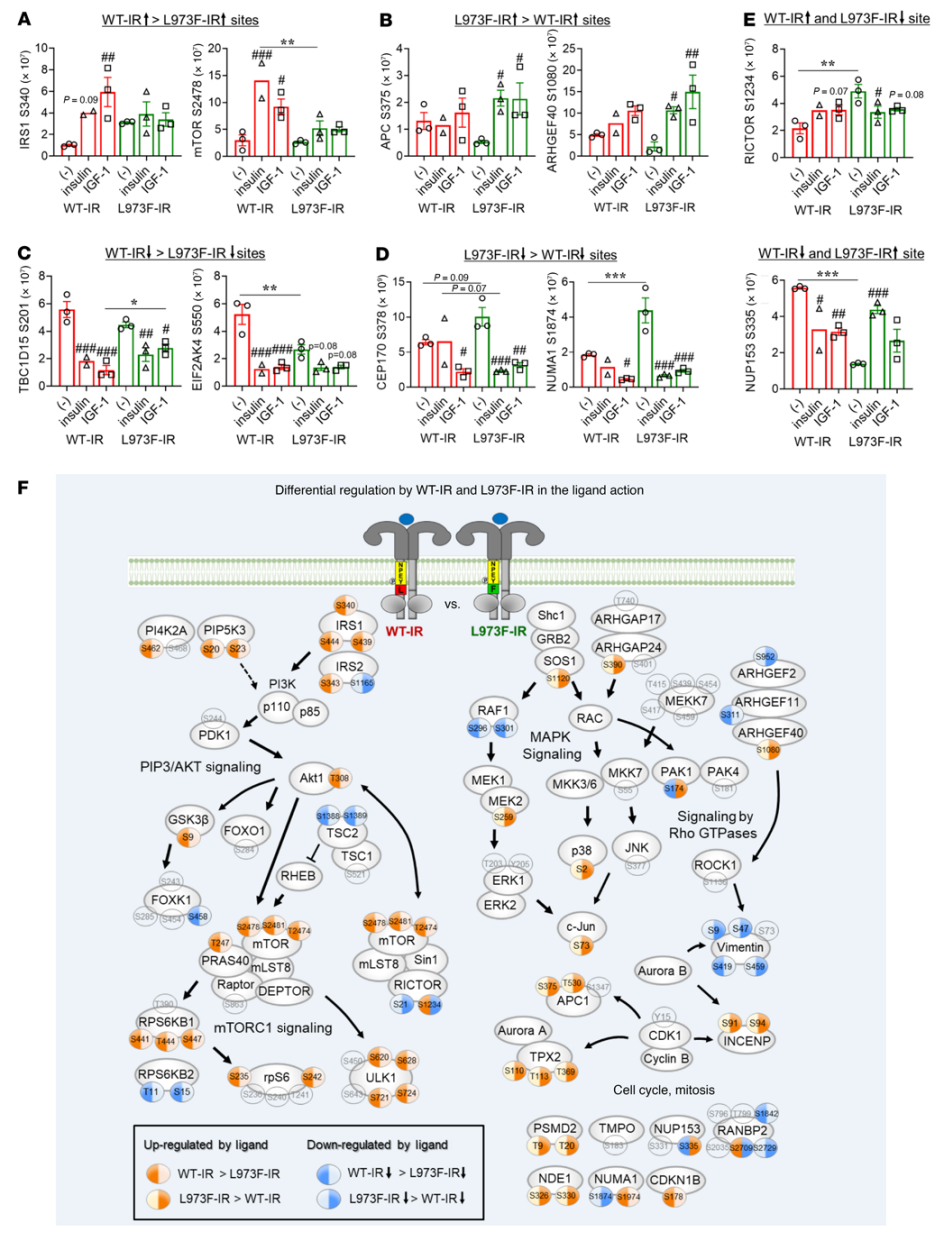
Integrated map showing differential signaling networks stimulated by the ligand-activated WT-IR versus L973F-IR preadipocytes. (**A**) Quantification of some important phosphosites upregulated by ligand (insulin or IGF-1) for fold-change WT-IR > L973F-IR. (**B**) Quantification of phosphosite examples upregulated by ligand (insulin or IGF-1) for fold-change L973F-IR > WT-IR. (**C**) Quantification of phosphosite examples downregulated by ligand (insulin or IGF-1) for fold-change L973-IR > WT-IR. (**D**) Quantification of phosphosite examples downregulated by ligand (insulin or IGF-1) for fold-change WT > L973F-IR. (**E**) Quantification of phosphosite examples shown opposite regulation by ligand between WT-IR and L973F-IR. Data are represented as mean ± SEM of phosphosites intensity values. ^#^*P* < 0.05; ^##^*P* < 0.01; ^###^*P* < 0.001 versus basal. *P < 0.05; **P < 0.01; ***P < 0.001, WT-IR versus L973F-IR, 2-way ANOVA. (**F**) Signaling map showing phosphosites unequally up- or downregulated by ligand stimulation of WT-IR and L973F-IR as identified by phosphoproteomics (*P* < 0.05). Sites with orange only on the left represent sites for which phosphorylation was upregulated more in WT-IR cells than L973F-IR cells following ligand stimulation. Sites with orange only on the right represent sites for which phosphorylation was upregulated more in L973F-IR cells than WT-IR cells following ligand stimulation. Sites with blue only on the left represent sites for which phosphorylation was downregulated more in WT-IR cells than L973F-IR cells following ligand stimulation. Sites with blue only on the right represent sites for which phosphorylation was downregulated more in L973F-IR cells than WT-IR cells following ligand stimulation. Arrows indicate known protein-protein interactions and phosphorylation/dephosphorylation events from databases (PhosphositePlus) and the literature.

To identify the full extent of the differential signaling responses to ligand stimulation between WT-IR and L973F-IR, we also compared the fold changes of each phosphosite between basal and stimulated conditions in these 2 cell types for both up- and downregulated phosphorylation in a volcano plot (Figure 3C). Of 354 phosphosites that were regulated by WT-IR to a greater extent than by L973F-IR (WT-IR > L973F-IR), 179 sites were upregulated by ligand stimulation (*P* < 0.05). Reactome pathway (https://reactome.org/) enrichment analysis of phosphosites upregulated more in WT-IR cells than in L973F-IR cells identified 6 pathways, including PIP3/Akt signaling and IR signaling pathways (Figure 3D). KEGG pathway analysis (https://www.genome.jp/kegg/pathway.html) also showed significant enrichment of proteins associated with the mTOR signaling pathway (Supplemental Figure 3, A and B). On the other hand, of 406 phosphosites that were more regulated by L973F-IR than by WT-IR (L973F-IR > WT-IR), 152 sites were upregulated by ligand stimulation (*P* < 0.05). Reactome pathway analysis of these phosphosites showed enrichment of pathways associated with cell cycle, mitosis, and many SUMOylation processes (Figure 3E). Quantitation of some of the differentially upregulated sites between WT-IR and L973F-IR is shown in Figure 4, A and B, and Supplemental Figure 3, B–D, including phosphorylation of IRS1^S340^ and mTOR^S2478^ in the PI3K/Akt signaling pathway and rpS6^S242^ and ULK1^S628^ in the mTOR signaling pathway, which were stimulated more in WT-IR cells than L973F-IR cells, while phosphorylation of APC1^S375^ and ARHGEF40^S1080^ were stimulated more in L973F-IR cells than WT-IR cells. Phosphosites associated with the MAPK activation pathway, such as c-Jun^S73^ and p38^S2^, were also upregulated more in L973F-IR cells than WT-IR cells (Supplemental Figure 3D).

Mass spectrometry analysis also identified 29 phosphosites that were differentially downregulated by ligand between WT-IR and L973F-IR (*P* < 0.05). Twenty-one of these sites were downregulated by ligand stimulation to a greater extent in WT-IR cells than L973F-IR cells, including TBC1D15^S201^ and EIF2K4S^550^, which are associated with intracellular trafficking and translational control (Figure 4C and Supplemental Figure 3F). On the other hand, 8 sites, including CEP170^S378^ and NUMA1^S1874^, were downregulated more in L973F-IR cells than WT-IR cells following ligand stimulation (Figure 4D and Supplemental Figure 3G). In addition, a few downstream phosphorylation sites showed opposite regulation by insulin between WT-IR and L973F-IR. For example, Ser1234 in RICTOR was upregulated by insulin in WT-IR cells, but downregulated by ligand in L973F-IR cells, while the converse was true for Ser355 in NUP153, a protein involved in nucleocytoplasmic transport of proteins and mRNAs (Figure 4E and Supplemental Figure 3H). Thus, substitution of Phe for Leu at position 973 in IR produces a complex series of alterations in signaling, in general, shifting the signaling pattern from metabolic pathways to mitogenic pathways.

To visualize the differential signaling events between WT-IR and L973F-IR, we generated an integrated signaling map of significant phosphorylation differences in signaling by WT-IR and L973F-IR (Figure 4F). Consistent with the above analyses, 3 phosphorylation sites on IRS-1 (IRS1^S340^, IRS1^S439^, IRS1^S444^) were preferentially stimulated by WT-IR compared with L973F-IR. Similarly, many phosphosites associated with mTORC1 signaling (mTOR^T2474^, mTOR^S2478^, mTOR^S2481^, RPS6KB1^S441^, RPS6KB1^T444^, RPS6KB1^S447^, S6^S235^, S6^S241^, and ULK1^S620^, ULK1^S628^, ULK1^S721^, ULK1^S724^) and several phosphosites related to PIP3-activated Akt signaling (Akt1^T308^, GSK3β^S9^, and PRAS^T247^) were more highly upregulated by WT-IR than L973F-IR. On the other hand, many phosphosites on proteins associated with cell cycle and mitosis (APC1^S375^, APC1^T530^, INCENP^S91^, INCENP^S94^, TPX2^S110^, TPX2^T113^, TPX2^T369^, PSMD2^T9^, PSMD2^T20^, and NDE1^S326^, NDE1^S330^) and several phosphosites associated with MAPK signaling (MEK2^S259^, p38^S2^, and c-Jun^S73^) were more highly upregulated by L973F-IR than WT-IR. Four phosphosites showed opposite regulation by ligand between WT-IR and L973F-IR. RICTOR^S1234^ and RANBP2^S2709^ were upregulated by ligand in WT-IR cells, but downregulated by ligand in L973F-IR cells, while the converse was true for PAK4^S174^ and NUP153^S335^. Several of these changes in the phosphoproteomics were confirmed by immunoblotting using phosphosite-specific antibodies, including phosphorylation of mTOR^S2481^ and S6^S235/236^, which were more highly upregulated by WT-IR than L973F-IR, while phosphorylation of c-Jun^S73^ was more highly upregulated by L973F-IR than WT-IR (Supplemental Figure 3, I and J). The latter agreed well with higher phosphorylation of JNK, an upstream kinase of c-Jun, in L973F-IR cells than in WT-IR cells (Supplemental Figure 3, K and L).

In addition to the phosphosites up- and downregulated by ligand stimulation, multiple proteins showed marked differences in basal phosphorylation in cells expressing the 2 receptor subtypes. Thus, phosphorylation of 32 sites on 20 proteins (category III-A) was not regulated by ligand, but was significantly higher in the basal state in cells expressing WT-IR versus L973F-IR, and phosphorylation of 140 sites on 109 proteins (category III-B) was significantly higher in the basal state in L973F-IR than in WT-IR cells. The former included GRB10^S455^ and FHDC1^S645^ and the latter IGF2BP2^S164^ and CCNE2^S21^ (Supplemental Figure 4, A and B). Since many ligand-regulated phosphosites were also different in basal phosphorylation between cells expressing these 2 receptors, we performed a heatmap analysis with hierarchical clustering focused only on basal phosphorylation (Supplemental Figure 4C). This revealed 236 sites for which basal phosphorylation was markedly higher in WT-IR cells than L973F-IR cells and 216 sites for which the converse was true (Supplemental Figures 4D). Pathway analysis of the proteins in the higher phosphorylation in WT-IR cluster showed enrichment of proteins involved in apoptosis, caspase-mediated cleavage, and signaling by RTKs (Supplemental Figure 4E). On the other hand, pathway analysis of the higher phosphorylation in L973F-IR showed enrichment in proteins involved in cell cycle (APC1^S377^, RANBP2^S2079^, and INCENP^S91,^ ^S94^), mitosis, kinesin action, and SUMOylation (Supplemental Figure 4F). Thus, in cells expressing the L973F-IR, phosphorylation of proteins related to the cell cycle and mitosis progression were enhanced in both the basal and ligand-stimulated states.

### Effects of the L973F substitution in the IR on gene transcription.

**Figure 5:**
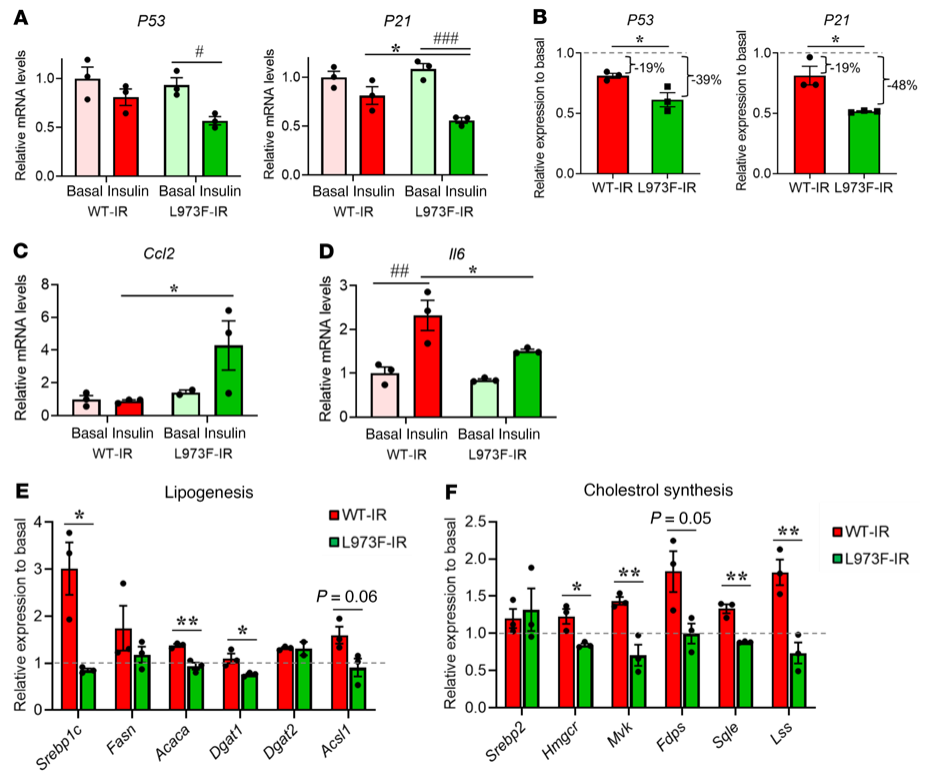
Effects of L973F substitution in IR on gene transcription. (**A**) Relative mRNA expression of *P53* and *P21* in WT-IR and L973F-IR cells. WT-IR and L973F-IR preadipocytes were stimulated with or without 100 nM insulin for 6 hours after 6 hours of FBS starvation. Gene expression levels of WT-IR cells at the basal were set at 1. (**B**) Fold changes of *P53* and *P21* expression in response to insulin stimulation in WT-IR and L973F-IR cells. (**C**) Gene expression levels of *Ccl2* in response to insulin stimulation in WT-IR and L973F-IR cells. (**D**) Gene expression levels of *Il6* in response to insulin stimulation in WT-IR and L973F-IR cells. (**E** and **F**) Fold changes of expression in response to insulin stimulation for genes associated with fatty acid synthesis (**E**) and cholesterol biosynthesis (**F**) in WT-IR and L973F-IR cells. Data are represented as mean ± SEM. ^#^*P* < 0.05; ^##^*P* < 0.01; ^###^*P* < 0.001, basal versus ligand. *P < 0.05; **P < 0.01, WT-IR versus L973F-IR, Student’s *t* test for fold change analysis and 2-way ANOVA for others.

Phosphoproteomic analysis clearly identified the MAPK/c-Jun signaling pathway as more activated in L973F-IR cells than in WT-IR cells by ligand stimulation, and this might be predicted to lead to increased cell proliferation in L973F-IR cells, since c-Jun signaling promotes G1-S transition and increases cell proliferation through repressing a p53-dependent pathway involving p53 and the Cdk-inhibitor p21 (27). Interestingly, while the mRNA levels of *P53* and *P21* were not different under basal conditions between WT-IR and L973F-IR preadipocytes, they were downregulated more in L973F-IR cells than in WT-IR cells after insulin stimulation for 6 hours (Figure 5, A and B). In agreement with higher MAPK/c-Jun activation in L973F-IR–expressing preadipocytes, mRNA expression of *Ccl2*, a downstream factor of c-Jun signaling (28), was also significantly induced by insulin only in L973F-IR preadipocytes (Figure 5C). On the other hand, the mRNA level of *Il6*, a downstream factor of PIP3/Akt/NF-κB signaling (29), was more upregulated by insulin in WT-IR preadipocytes than in L973F-IR preadipocytes (Figure 5D).

Insulin is known to modulate many metabolic genes. For example, insulin stimulation increases the expression of genes regulating fatty acid and cholesterol biosynthesis, whereas insulin deficiency impairs this upregulation (30). Consistent with the lower degree of metabolic signaling by the L973F-IR, stimulation of many genes related to fatty acid synthesis (including *Srebp1c* and *Acaca*) and cholesterol synthesis (such as *Hmgcr*, *Mvk*, and *Fdps*) by insulin in WT-IR preadipocytes was severely blunted in L973F-IR preadipocytes (Figure 5, E and F, and Supplemental Figure 5, A and B). Similar findings were observed in the adipocytes after differentiation (Supplemental Figure 5, C–F). Stimulation of genes related to fatty acid synthesis and cholesterol synthesis by insulin in differentiated adipocytes was also severely blunted in L973F-IR–differentiated adipocytes. Thus, signaling alterations by L973F substitution in the IR decreases the anabolic effects of insulin on gene transcription associated with fatty acid synthesis and cholesterol biosynthesis while increasing the mitogenic effects on gene transcription involved in control of cell cycle progression.

### Effects of L973F substitution on IR signaling in mice.

**Figure 6:**
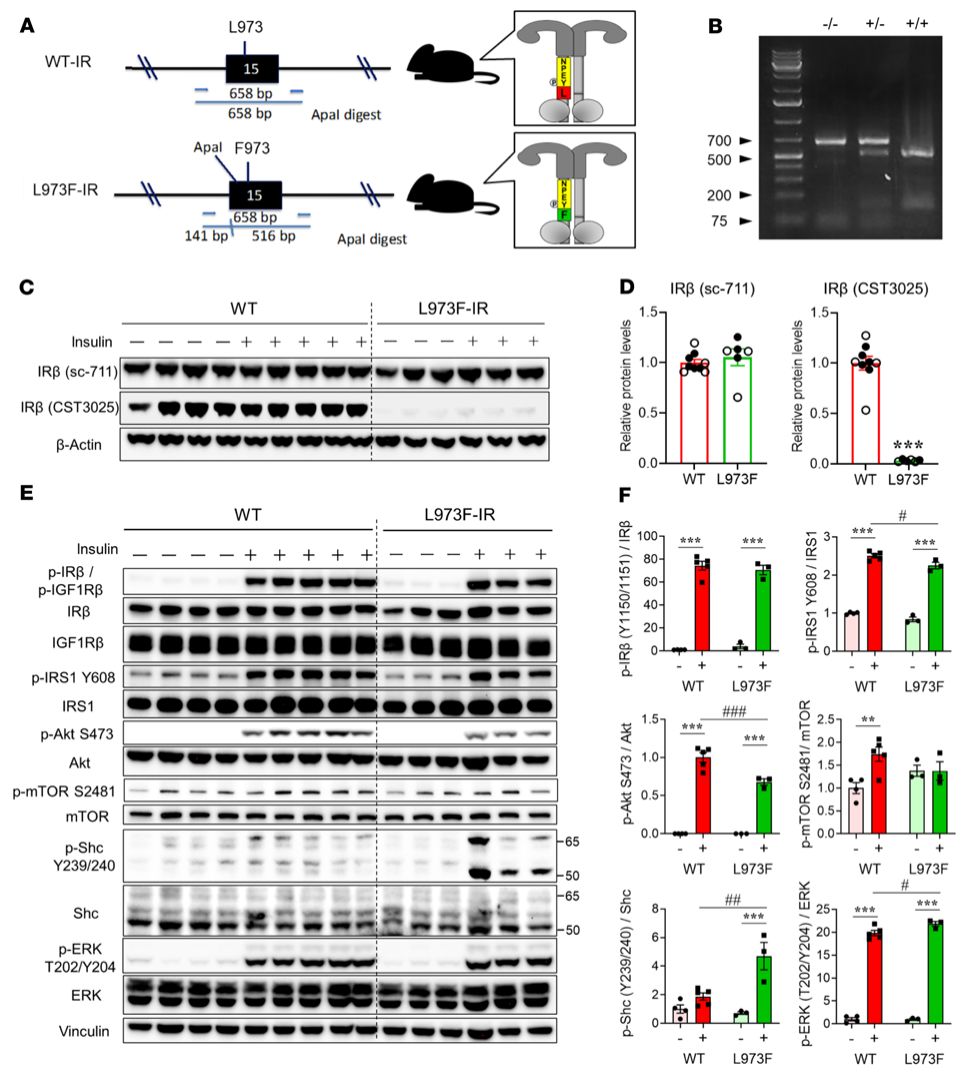
Effects of the L973F substitution on IR signaling in mice. (**A**) Schematic representation of L973F-IR modification in the genome of C57BL/6N mice by CRISPR-mediated gene editing. There were also 2 silent point mutations inserted in codons for glycine-962 and proline-963 to generate a new ApaI restriction site for genotyping. (**B**) Genotyping analysis of L973F mutation knockin mice. WT mouse (–/–) produces a 658 bp fragment. Homozygous knockin mouse (+/+) produces 516 bp and 141 bp fragments. Heterozygous knockin mouse (+/–) produces 658 bp, 516 bp, and 141 bp fragments. (**C**) IR levels in liver tissue from male WT and L973F-IR mice (7 months old) by immunoblotting using antibodies specific for IRβ from Santa Cruz Biotechnology Inc. (sc-711) and Cell Signaling Technology (CST3025). Mice were injected with either saline or 2 units insulin via the vena cava 10 minutes before sacrifice. CST3025 detects residues surrounding tyrosine-972 in the juxtamembrane region of IRβ. SC-711 antibody detects the C-terminus of IRβ. (**D**) Quantification of IRβ levels in liver tissues from male WT and L973F-IR mice. The level of each protein in the control mice was set at 1. Saline-injected mice are represented by white circles and insulin-injected mice by black circles. Data are represented as mean ± SEM (*n* = 6–9 per group). ***P < 0.001, WT versus L973F-IR mice, unpaired Student’s *t* test. (**E**) Analysis of insulin signaling in gastrocnemius muscle of WT and L973F-IR mice (7-month-old males) administered insulin (2 U) or saline 10 minutes prior to sacrifice. Immunoblot analysis was performed with the indicated antibodies. (**F**) Quantification of p-IRβ, p-IRS1Y608, p-AktS473, p-mTORS2481, p-ShcY239/240, and p-ERKT202/Y204. Results are represented as mean ± SEM. **P < 0.01; ***P < 0.001, basal versus insulin. ^#^*P* < 0.05; ^##^*P* < 0.01; ^###^*P* < 0.001, WT versus L973F-IR mice, 2-way ANOVA.

To determine the full effects of the L973F substitution on IR function in vivo, we generated a knockin mouse in which the L973F substitution was introduced into the endogenous INSR locus using CRISPR/Cas9 mutagenesis (Figure 6A). We also introduced silent point mutations at the codons for glycine-962 and proline-963 to generate a new ApaI restriction site to simplify genotyping. Successful introduction of the mutation was confirmed by sequencing (Supplemental Figure 6) and genotyping (Figure 6B). These changes produced no changes in fetal viability, allowing use of homozygous L973F-IR mice for further analysis. Western blotting of liver extracts from adult mice using the IRβ antibody directed at the C-terminus (Santa Cruz Biotechnology Inc., sc-711) confirmed equal levels of IR in WT and L973F-IR mice (Figure 6, C and D). In contrast, Western blotting using an IRβ antibody directed at the sequence surrounding tyrosine-972 in IRβ (CST3025) revealed a strong signal in livers of mice with the WT-IR, but almost no detectable signal in livers of mice expressing L973F-IR.

To understand how this change in the IR affects insulin signaling in vivo, mice were fasted 6 hours and then injected with insulin or saline via the inferior vena cava and sacrificed 10 minutes later. Consistent with the in vitro analysis, insulin stimulation induced robust and equal autophosphorylation of IRβ in skeletal muscle of both WT and L973F-IR mice. At the postreceptor level, however, mice with the WT receptor showed significantly higher phosphorylation of IRS-1^Y608^, Akt^S473^, and mTOR^S2481^ than L973F-IR mice (Figure 6, E and F). Conversely, and even more strikingly, the L973F-IR mice displayed an almost 3-fold increase in Shc phosphorylation as well as a small, but significant, increase in ERK phosphorylation (Figure 6, E and F). Similar differences in phosphorylation were observed in other tissues. Thus, compared with mice with WT-IR, in L973F-IR mice, insulin-dependent phosphorylation of Akt was reduced and the phosphorylation of Shc was increased in white adipose tissue (WAT) (Supplemental Figure 7, A and B) and liver (Supplemental Figure 7, C and D).

**Figure 7:**
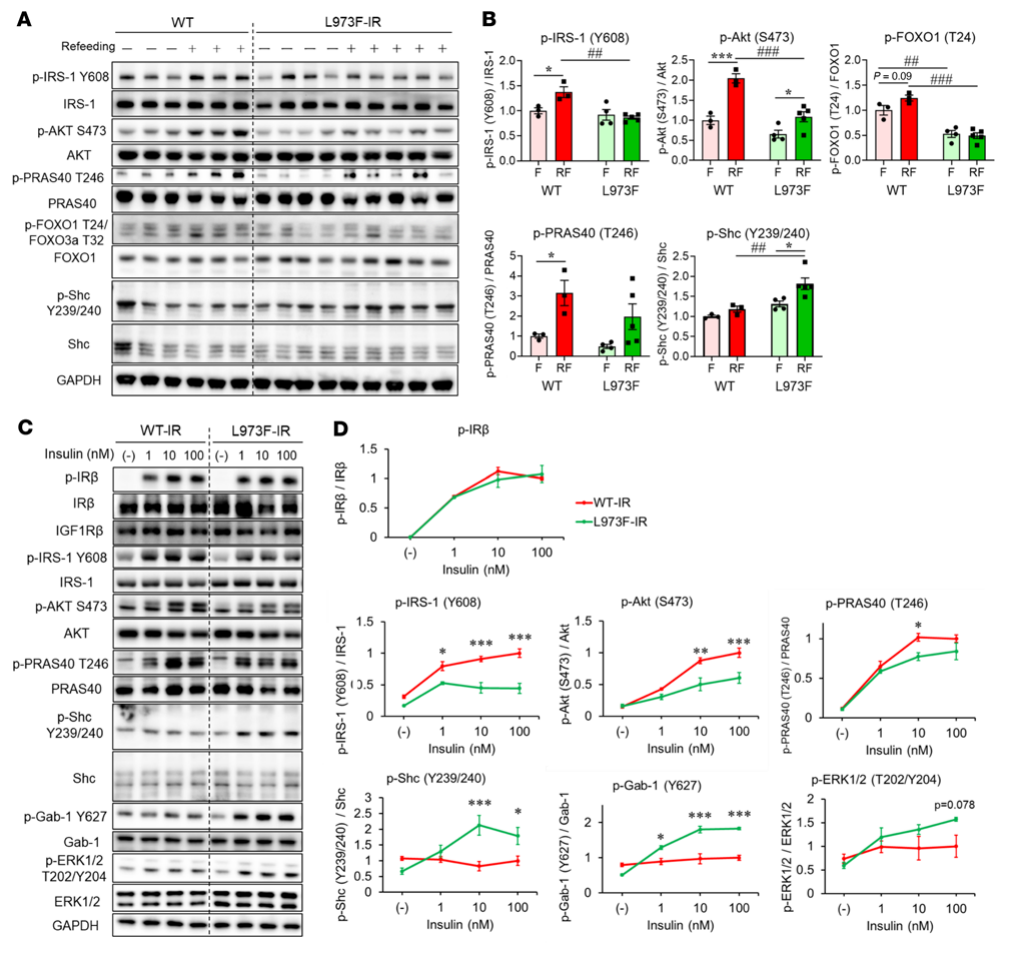
Effects of L973F substitution on IR signaling in fasting/refed mice and primary cells. (**A**) Immunoblot analysis of insulin signaling in gastrocnemius muscle of WT and L973F-IR mice (6-month-old males) starved overnight (15 hours) and then refed for 2 hours, followed by sacrifice and tissue harvest. (**B**) Quantification of p-IRS1^Y608^, p-Akt^S473^, p-Foxo1^T24^, p-PRAS40^T246^, and p-Shc^Y239/240^. The level of each phosphorylated protein in WT fasting mice was set at 1. Data are represented as mean ± SEM. *P < 0.05; ***P < 0.001, fasting, not refed (F) versus refed (RF). ^##^*P* < 0.01; ^###^*P* < 0.001, WT versus L973F-IR mice, 2-way ANOVA. (**C**) Immunoblot analysis of insulin signaling in primary hepatocytes from WT and L973F-IR mice stimulated with 0, 1, 10, or 100 nM insulin for 15 minutes. (**D**) Densitometric analysis of phosphorylated proteins following insulin stimulation. The level of each phosphorylated protein in cells from WT mice stimulated with 100 nM insulin was set at 1. Data are represented as mean ± SEM (*n* = 3). *P < 0.05; **P < 0.01; ***P < 0.001, WT-IR versus L973F-IR, 2-way ANOVA.

To understand these differences in receptor signaling under more physiologic conditions, we assessed these signaling pathways in mice after overnight fasting and 2 hours of refeeding. Mice with the WT receptor showed significantly higher phosphorylation of IRS-1^Y608^ and Akt^S473^ than L973F-IR mice (Figure 7, A and B). Phosphorylation of Foxo1 and PRAS40, downstream targets of Akt, showed similar patterns. Conversely, the L973F-IR mice displayed an increase in Shc phosphorylation in muscle in vivo (Figure 7, A and B). Similar differences in phosphorylation were observed in the liver and WAT. Thus, compared with mice with WT-IR, in L973F-IR mice, insulin-dependent phosphorylation of Akt was reduced and the phosphorylation of Shc was increased in the liver (Supplemental Figure 8, A and B) and WAT (Supplemental Figure 8, C and D). These findings under the physiological conditions were consistent with the results observed following vena cava insulin injection.

To further clarify the differences of dose response in insulin signaling, primary hepatocytes were isolated from WT-IR and L973F-IR mice and stimulated with 0, 1, 10, and 100 nM insulin for 15 minutes. There were no changes in the normal levels of endogenous IGF1R in this knockin model, and phosphorylation of the receptors in cells from WT and L973F-IR mice was similar (Figure 7, C and D). As with the DKO cells engineered in vitro, cells from the in vivo L973F-IR knockin mice showed decreased responsiveness to insulin stimulation in phosphorylation of IRS-1^Y608^, Akt^S473^, and PRAS40^T246^ compared with the cells from WT mice (Figure 7, C and D). On the other hand, insulin-stimulated Shc, Gab-1, and ERK phosphorylation were increased in the hepatocytes from L973F-IR (Figure 7, C and D). Together, these findings indicate that the L973F substitution in IR alters downstream receptor signaling, favoring a mitogenic compared with a metabolic response both in vitro and in vivo.

### Effects of the L973F substitution on metabolism and growth in mice.

**Figure 8:**
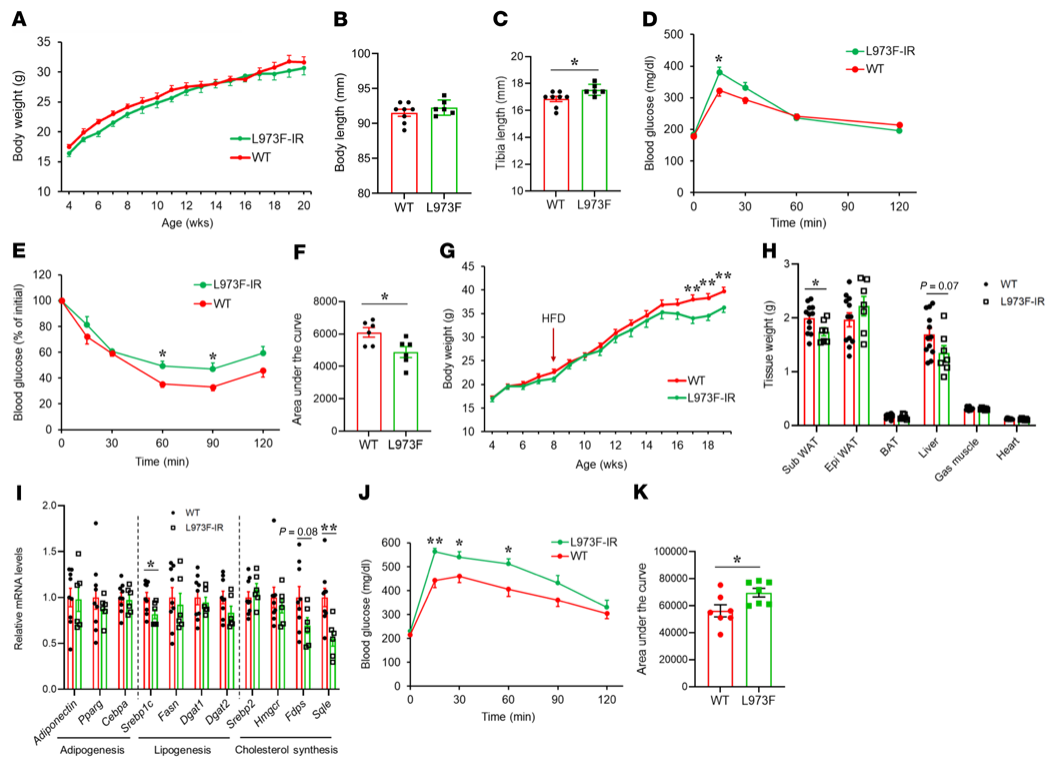
Effects of L973F substitution on metabolism and growth in mice. (**A**) BW of WT and L973F-IR male mice measured over the indicated time course. All mice were fed a normal chow diet (*n* = 6–8). (**B**) Body length of 7-month-old male chow-fed mice (*n* = 6–8). (**C**) Tibial length as measured by dual x-ray absorptiometry (DEXA) scan in 21-week-old male chow-fed animals (*n* = 6–8). (**D**) IPGTTs were performed at 16 weeks of age in male chow-fed animals (*n* = 6–8) as described in Methods. (**E**) ITTs were performed in 14-week-old male chow-fed mice as described in Methods (*n* = 6). (**F**) Area over the curve of blood glucose during ITT. (**G**) BWs of WT and L973F-IR male mice were measured over the indicated time course. These mice were fed an HFD from 8 weeks of age for 3 months (*n* = 8-10). (**H**) Tissue weights of 5-month-old HFD-fed male mice (*n* = 7–12). Epi WAT, epididymal WAT; BAT, brown adipose tissue; Gas muscle, gastrocnemius muscle. (**I**) Changes in mRNA levels of genes associated with adipogenesis, lipogenesis, and cholesterol synthesis in Sub WAT of WT and L973F-IR HFD–fed male mice (*n* = 6–9). (**J**) IPGTT in HFD-fed male mice at 19 weeks of age (*n* = 7). (**K**) Area under the curve of blood glucose during IPGTT in HFD-fed male mice. Data are represented as mean ± SEM. *P < 0.05; **P < 0.01, WT versus L973F-IR mice, unpaired Student’s *t* test.

At the physiological level, on normal chow diets, there was no significant difference in body weight (BW) between male WT and L973F-IR mice, at least over the first 5 months of life (Figure 8A). Both types of mice showed comparable food intake, lean body mass, total fat mass, and tissue weights (Supplemental Figure 9, A–D). L973F-IR knockin mice, on the other hand, showed a trend toward an increase in body length (Figure 8B), and tibial length was significantly increased compared with that in controls (Figure 8C). Fasting plasma glucose and insulin levels were not significantly different between WT and L973F-IR mice (Supplemental Figure 9, E and F). However, L973F-IR mice did display slightly impaired glucose tolerance with significantly higher glucose levels at 15 minutes after glucose injection compared with controls (Figure 8D). In addition, L973F-IR mice showed a significant impairment in the glucose-lowering effect of exogenous insulin during an insulin-tolerance test (ITT) with an approximately 20% decrease in area over the curve (Figure 8, E and F). On pyruvate-tolerance testing (PTT) to assess gluconeogenesis, the blood glucose levels after pyruvate injection were significantly higher in L973F-IR mice compared with WT mice, indicating an increase in hepatic glucose production in L973F-IR mice (Supplemental Figure 9, G and H). Female mice showed changes similar to those of male mice, with no significant differences in BW, tissue weight, or tibial length between WT and L973F-IR mice (Supplemental Figure 10, A–D), but a significant increase in total body length in L973F-IR mice compared with controls (Supplemental Figure 10E), mild glucose intolerance (Supplemental Figure 10F), and significantly impaired insulin sensitivity, as assessed by ITT (Supplemental Figure 10, G and H).

To further explore the effects of the L973F substitution in the IR in obesity, both male WT and L973F-IR knockin mice were challenged with HFD. As in the chow-fed mice, vena cava injection of insulin triggered a smaller induction of phosphorylation on IRS-1^Y608^ and Akt^S473^ in skeletal muscle of L973F-IR mice compared with WT control mice (Supplemental Figure 11, A and B). Likewise, the phosphorylation of Shc and Gab-1 were increased by more than 2-fold in L973F-IR mice compared with controls while on HFD (Supplemental Figure 11, A and B). Interestingly and importantly, however, when challenged with HFD, the L973F-IR knockin mice displayed significantly lower BW gain than controls (Figure 8G and Supplemental Figure 11C), with no changes in food intake (Supplemental Figure 11D). L973F-IR mice also had a significant reduction in the mass of subcutaneous WAT (Sub-WAT) than controls and a trend toward a lower liver weight, but had epididymal white fat mass and interscapular brown fat mass similar to that of control mice on HFD (Figure 8H). The reduced mass of Sub-WAT in the HFD-fed L973F-IR mice was associated with lower expression of genes related to lipogenesis and cholesterol synthesis, including *Srebp1c* and squalene monooxygenase (*Sqle*) (Figure 8I). We also evaluated hepatic genes related to lipid and cholesterol metabolism. *Srebp1c* tended to be decreased in livers of L973F-IR mice, but many of the other measured lipogenic genes were not significantly different between WT and L973F-IR mice (Supplemental Figure 11E). On the other hand, many genes related to cholesterol synthesis were decreased in livers of L973F-IR mice (Supplemental Figure 11E), as with the findings in WAT. Consistent with this, L973F mice displayed decreases in hepatic and plasma cholesterol levels (Supplemental Figure 11, F and G). The liver triglyceride levels also tended to be lower in L973F-IR mice, but this was not significant, and there were no differences in the plasma triglyceride levels (Supplemental Figure 11, H and I). HFD-challenged L973F-IR mice, on the other hand, showed no differences in body length or tibial length (Supplemental Figure 11, J and K) and no significant differences in fasting plasma glucose and insulin levels from WT mice (Supplemental Figure 11, L and M). Consistent with the data on normal chow, however, L973F-IR mice displayed significantly impaired glucose tolerance, as assessed by intraperitoneal glucose-tolerance test (IPGTT), compared with controls (Figure 8, J and K), despite their lower BW. Indeed, this difference in glucose intolerance was even greater than that in the normal chow diet–fed mice. HFD-fed L973F-IR mice showed slightly higher glucose levels during an ITT than WT-IR mice (Supplemental Figure 11N). The higher levels of insulin resistance in HFD-fed obese WT mice likely make it more difficult to find a difference in insulin resistance due to L973F substitution under these conditions. Together, these findings suggest reduced insulin action on metabolism, i.e., increased insulin resistance, despite the lower gain in BW and fat mass. Thus, the L973F-IR knockin mice challenged with HFD showed altered signaling with the switch from metabolic to growth dominance and worsening of glucose intolerance, despite protection from diet-induced obesity.

## Discussion

Insulin and IGF-1 are major regulators of metabolism and systemic growth both in vivo and in vitro (1, 5). Thus, insulin acts to promote uptake and storage of glucose and other fuels, while IGF-1 is a potent stimulator of mitogenesis and organismal growth (8, 9, 12, 14, 15). Since the discovery that these hormones act through highly homologous receptors with highly homologous postreceptor signaling pathways, one of the big challenges in the field has been to understand how these two hormones produce such distinct biological effects with such similar mechanisms of signal transduction. Some differences between these receptors in substrate phosphorylation have been observed (14), and recently, using a global phosphoproteomics approach, we have demonstrated that IR and IGF1R have both distinct and overlapping patterns of signaling (15). Indeed, many phosphosites associated with PI3K/Akt signaling, mTOR signaling, and membrane trafficking are preferentially upregulated by ligand stimulation of the IR, whereas IGF-1 stimulation of IGF1R favors phosphorylation of proteins associated with Rho-GTPases, cell cycle, and mitosis. Clinically, insulin resistance and altered IR signaling is linked to diseases such as diabetes, accelerated atherosclerosis, and fatty liver disease (31–34), whereas IGF1R mutations or IGF-1 deficiency cause pre- and postnatal growth retardation and specific defects in tissue growth, e.g., microcephaly (35, 36). On the other hand, excess signaling through IGF1R can contribute to gigantism and cancer progression (37).

Several studies have shown that the NPEY motifs in the juxtamembrane region of the IR and IGF1R have key roles in the recruitment of the immediate substrates of IR, including IRS-1/2 and Shc (5, 16, 17). We have previously shown that the residue C-terminal to the NPEY motif is also important in substrate binding. Thus, replacement of leucine-973 at position +1 after the NPEY motif of IR to phenylalanine, which is the amino acid at the equivalent position in the IGF1R, decreases IRS-1 binding and markedly increases Shc binding (14). Conversely, substitution of leucine-973 by alanine or arginine severely reduces Shc binding with little effect on IRS-1 binding (38). In the present study, we have explored the role of this difference in the IR and IGF1R in vitro and in vivo by characterization of cells and mice in which the IR has been replaced with a mutated IR in which leucine-973 of IR has been replaced by phenylalanine.

We find that in cells, this results in a reduction in IRS-1/PI3K/Akt/mTORC1-mediated signaling with even more marked upregulation of Shc/Gab1/MAPK signaling and phosphorylation of cell cycle–related proteins. Global phosphoproteomics analysis also reveals the differences in signaling by L973F substitution, including decreased phosphorylation of proteins involved in the SUMOylation process and increased phosphorylation of proteins involved in apoptotic signaling and mRNA splicing. These signaling alterations associated with the L973F-IR result in decreased insulin-induced glycolysis and increased cell growth rate in L973F-IR cells. These data are in agreement with our recent phosphoproteomic analysis of the IR versus the IGF1R themselves (15). Thus, many phosphosites associated with mTORC1 signaling in the WT-IR > L973F-IR cluster also showed higher phosphorylation in IR compared with IGF1R-expressing cells, while some of the phosphosites associated with cell cycle in the L973F-IR > WT-IR cluster were also upregulated in IGF1R-expressing cells (15). Thus, while not the only factor, this single amino acid difference is a major determinant of the differential signaling between IR and IGF1R.

In addition to substrate binding, the NPEY motif in both IR and IGF1R is known to be involved in receptor internalization (20, 22). Previous studies have shown that internalization of these receptors following ligand stimulation is more pronounced in IR than IGF1R (14), and this effect is decreased in insulin-resistant obese subjects and obese type 2 diabetic patients (24). Consistent with its more IGF1R-like behavior, we find that the L973F mutation in IR leads to decreased receptor internalization following insulin stimulation compared with WT-IR.

In addition to the insulin-stimulated differences in protein phosphorylation, many phosphosites detected in the phosphoproteomics are altered under basal conditions by changing leucine at position 973 to phenylalanine. Indeed, many of the phosphosites upregulated by L973F-IR in the basal state were on proteins associated with cell cycle and mitosis. We have previously shown that, in addition to its classical signaling pathway, the unoccupied IR can exert some ligand- and tyrosine kinase–independent effects (39, 40). For instance, preadipocytes lacking both IR and IGF1R (DKO) display resistance to apoptosis, and this resistance to apoptosis is rescued by reexpression of both WT-IR and, more importantly, a kinase-inactivated IR (K1030R-IR) (40). We have found that there are also many differences in the phosphoproteome between cells expressing IR versus IGF1R in the basal state (unstimulated by ligand), including phosphorylation of proteins involved in cell cycle and mitosis (15). These are very similar to those observed with the L973F-IR receptor, indicating the importance of this site in both basal and ligand-dependent signaling. Further research using the substitution of phenylalanine to leucine in the IGF1R would be needed to understand the importance of the difference in amino acid in the +1 position following the NPEY motif on IGF1R signaling.

To fully explore the role of the L973F mutation of the IR, we created a knockin mouse in which the normal receptor was replaced by this mutated receptor in all tissues. Previous studies have shown that enhanced IGF-1 signaling can result in increases in tissue weight and somatic growth (41–45). For example, IGF-1 overexpression in liver increases BW, lean mass, and body length (41), and IGF-1 overexpression in muscle or heart leads to hypertrophy and localized regeneration (43–45). Likewise, overexpression of IGF1R in heart induces cardiac hypertrophy (42). In the present study, we generated a knockin mouse in which the L973F substitution was introduced into the endogenous INSR locus. This mouse model is more physiological than the mouse model with overexpressing receptors, since the receptor level is under the normal promoter control. We found that homozygous knockin of the L973F-IR in mice results in alterations in signaling similar to those observed in vitro, i.e., decreased IRS-1–mediated signaling and increased Shc-mediated signaling, and these lead to impaired insulin sensitivity and modest increases in either tibial or whole-animal growth. Interestingly, and perhaps more importantly, mice carrying the L973F- IR also showed a decrease in weight gain and reduced size of Sub-WAT following challenge with HFD. This could reflect the effects of the decrease in IRS-1–mediated insulin signaling in adipose or a difference in the balance of insulin/IGF-1 action on adipocyte differentiation (46).

While the L973F-IR shifts the signaling pattern from a more metabolic to a more mitogenic pattern, the effects of L973F-IR on growth in vivo were modest. This may be due to the existence of an intact IGF1R in both WT and knockin mice as well as effects from both ligands, insulin and IGF-1, which could at least partially compensate for their downstream signaling effects. Some in vivo studies have shown that blocking both insulin and IGF-1 downstream signaling by deletion of both IR and IGF1R result in obvious phenotypes, which are not obvious in single deletion of IR or IGF1R (47, 48). For example, muscle mass shows only a small reduction by muscle-specific single deletion of either IR or IGF1R, but is markedly decreased by muscle-specific deletion of both IR and IGF1R (47).

Altered downstream signaling by the IR with the L973F substitution is certainly in large part mediated by the decrease in IRS-1 binding/phosphorylation and increase in Shc binding/phosphorylation, as shown in this study. We have previously shown that this is based on the differences in structure of the SH domains of these two different receptor substrates (14). This structure feature could cause decreased responsiveness to insulin in metabolic downstream signaling even in high receptor occupancy of L973F-IR. Further studies of other docking proteins and key kinases involved in this differential signaling would be needed to understand the relevance of the difference in amino acid in the +1 position following the NPEY motif on signaling. In addition, the L973F substitution does not fully explain all the differences between IR and IGF1R signaling. Further studies to understand the role of other sequence differences in the intracellular domains of IR and IGF1R that may also contribute to their specific physiological effects are warranted to fully understand how these two highly homologous receptors produce such distinct biological effects.

In summary, we have demonstrated that a single amino acid replacement of leucine-973 in the juxtamembrane region of IR to phenylalanine, which is the equivalent residue in the IGF1R, is sufficient to lead to a decrease of IRS-1/PI3K/Akt/mTORC1 signaling and an increase of Shc/Gab1/MAPK cell cycle signaling. At the cellular level, these signaling alterations lead to decreases in insulin-induced glycolysis and increases in cell growth. Mice with knockin of the L973F-IR show similar alterations in signaling in vivo, and this leads to impaired insulin sensitivity, modest increases in growth, and decreases in weight gain on HFD. Thus, leucine-973 in the +1 position following the NPEY motif in the juxtamembrane region is a key residue differentiating IR signaling from IGF1R signaling. This study explains a big part of the differences between IR and IGF1R signaling and may provide important insights into how to modify these signaling pathways for therapy of disease.

## Methods

### Materials.

Antibodies against phosphorylated IRβ (p-IRβ)/IGF1Rβ (catalog 3024), IRβ (catalog 3025), IGF1Rβ (catalog 3027), β-actin (catalog 4970), GAPDH (catalog 5174), p-AKT^S473^ (catalog 4060), AKT pan (catalog 4685), p-ERK1/2^T202/Y204^ (catalog 4370), ERK1/2 (catalog 9102), p-Shc^Y239/240^ (catalog 2434), Shc (catalog 2432), p-Gab-1^Y627^ (catalog 3233), Gab-1 (catalog 3232), p-mTOR^S2481^ (catalog 2974), mTOR (catalog 2972), p-c-Jun^S73^ (catalog 3270), c-Jun (catalog 9165), p- p-S6^S235/236^ (catalog 2211), S6 (catalog 2217), p-JNK^T183/Y185^ (catalog 4668), and JNK (catalog 9252) were purchased from Cell Signaling Technology. Anti-IRβ antibody (catalog sc-711) was purchased from Santa Cruz Biotechnology Inc. Anti–p-IRS-1 (catalog Y608) antibody (catalog 09-432), anti–IRS-1 antibody (catalog 06-248), and anti-vinculin antibody (MAB3574) were purchased from MilliporeSigma. Human insulin was purchased from MilliporeSigma. Human IGF-1 was purchased from PeproTech. The human IR (B isoform) retroviral plasmid was generated previously in the lab (49). L973F-IR (aa numbers excluding signal peptide) were generated from human IR (B isoform) cDNA using a site-directed mutagenesis kit from Agilent. Primers for L973F-IR generation were 5′-ACGCTTCTTCAAACCCTGAGTATTTCAGTGCCAGTG-3′ and 5′-CACTGGCACTGAAATACTCAGGGTTTGAAGAAGCGT-3′. For coimmunoprecipitation assays, WT human IR and L973F-IR cDNA were cloned into 3_Flag-CMV-14 mammalian expression vector (MilliporeSigma).

### Brown preadipocyte isolation, retroviral infection, and culture.

IR/IGF1R DKO brown preadipocytes were obtained as previously described (15, 40). DKO preadipocytes were then stably transduced with pBabe retrovirus containing the cDNA for the human IR (B isoform) or the L973F-IR. Plates (10 cm) of Phoenix cells were transiently transfected with 10 μg of pBabe-hygro retroviral expression vectors encoding WT, L973F-IR, and IGF1R sequences. Methods after transfection were as previously described (15).

### CRISPR-mediated generation of a knockin mouse with the INSR L973F.

Oocyte isolation from C57BL/6 and pronucleus injections were performed at the Transgenesis Core Facility (TCF) of the Max Planck Institute (MPI) for Biology of Ageing. To prepare gRNAIR, 5 μg crRNA (GCTCACCATCACTGGCACTG) was mixed with 10 μg tracrRNA heated to 95°C for 5 minutes and then cooled down to room temperature. To exchange codon CTC of the IR encoding leucine-973 to codon TTC encoding phenylalanine-973, we performed pronucleus injections. Injection solution was prepared as follows: 20 ng/μl gRNA-IR was incubated with 20 ng/μl Cas9 protein for 15 minutes at room temperature to generate the ribonucleotide complex RNP. Subsequently, a single stranded repair template that had 100 bp homologous regions 5′ and 3′ to the modifications was added at 20 ng/μl (AAATGTCGAACAATCCATTCCTAATTTATATAGCATTTGCCTATGTCCAGAATGCCATGGGATGTTCAAGATGTTGTGTCTCTCTTCCTCCATTAGGCAGCCGGATGGGCCAATGGGGCCCCTGTATGCATCTTCAAACCCTGAGTAttTCAGTGCCAGTGATGGTGAGCATCACCTCCTTCTTTGTGGGAATCCAGAACCCAGCCCTTGGTTTTCTTTGCTATCACTGTTAAGTCAGATCTGAGGATAGGCTAGCATCACAGGG). All reagents were obtained from IDT. gRNA-mediated Cas9 double-strand break of genomic DNA between codons tyrosine-972 and leucine-973 should initiate homologous-directed repair using the ssDNA repair oligo, thereby mutating the PAM sequence, exchanging codon leucine-973 to phenylalanine-973, as well as inserting silent point mutations at codons glycine-962 and proline-963 to generate an ApaI restriction site for genotyping. Offspring were genotyped with a common PCR reaction using primers 5′-CCCTCACTTCCTCTAATTTGGACA-3′ and 5′-CACTATGTAAAGTCAAACCCAGT-3′ to result in a 658 bp fragment for both WT and L973F-IR mice. Subsequent restriction digest of PCR fragments with ApaI resulted in 141 bp and 516 bp fragments when positive for the F973 modification, whereas nonmodified WT DNA did not contain the ApaI site and thus still showed a 658 bp band upon ApaI digestion. PCR products from offspring were subcloned and Sanger sequenced and confirmed the correct codon L973F exchange in IR exon 15 as well as the ApaI site at glycine-962/proline-963. All mice were kept on a C57BL/6N background and housed at 22°C on a 12-hour-light/12-hour dark cycle with ad libitum access to water and food (Mouse Diet 9F, PharmaServ). HFD (60% fat, 20% protein, and 20% carbohydrates) was purchased from Research Diets (D-12492). For the intraperitoneal ITT, mice were fasted for 6 hours and injected with insulin at 1 U/kg/BW for male mice fed a normal diet, 0.8 U/kg/BW for female mice fed a normal diet, or 1.6 U/kg BW for male mice fed HFD. For the IPGTT, mice were fasted for 6 hours before injection of glucose at 2 g/kg/BW. For the intraperitoneal PTT, mice were fasted overnight before injection of pyruvate at 2 g/kg/BW.

### Quantification of mRNA levels by qPCR.

Total RNA was isolated from cells using TRIzol reagent (Thermo Fisher Scientific). cDNA was synthesized and then quantitative PCR (qPCR) amplification was conducted with the C1000 Thermal Cycler (Bio-Rad, catalog CFX384) using iQ SYBR Green Supermix (Bio-Rad). The sequences of primers were as follows: human *INSR*, 5′-CGATATGGTGATGAGGAGCTGC-3′ and 5′-GTAGAAATAGGTGGGTTCCGTCCA-3′. Mouse primers used for qPCR are listed in Supplemental Table 1. Expression levels of specific genes were normalized to TBP or 36B4.

### Insulin signaling.

WT-IR and L973F-IR preadipocytes were FBS starved for 6 hours with DMEM containing 0.1% BSA, then stimulated with insulin for 15 minutes. After stimulation, cells were washed once with ice-cold PBS and collected in RIPA buffer (MilliporeSigma) supplemented with protease and phosphatase inhibitors (BioTools). Cell lysates were then centrifuged at 20,000*g* for 15 minutes at 4°C and supernatants were collected. Protein concentrations were measured using the Pierce BCA Protein Assay Kit (Thermo Fisher Scientific).

### Immunoblotting.

Proteins were transferred to polyvinylidene difluoride (PVDF) membranes. Membranes were then blocked in Starting Block T20 solution (Thermo Fisher Scientific) at room temperature for 1 hour, washed 3 times with PBS with Tween-20 (PBST), and incubated with an appropriate dilution of the primary antibody (all antibodies were used at a dilution of 1:1,000 except for GAPDH and β-actin, which were used at dilutions of 1:5,000) in Starting Block T20 solution overnight at 4°C. Membranes were washed with PBST and incubated with an appropriate dilution of secondary antibody in Starting Block T20 solution for 1 hour. Sheep anti-mouse HRP-conjugated antibody (catalog NA931) was purchased from MilliporeSigma, and goat anti-rabbit HRP-conjugated antibody (catalog 1706515) was purchased from Bio-Rad. Membranes were washed with PBST, and signals were detected using chemiluminescent HRP substrates (Thermo Fisher Scientific).

### Ligand-induced glycolysis and adipocyte differentiation.

The glycolysis rate of WT-IR and L973F-IR preadipocytes was measured by Seahorse XFe96 Bioanalyzer (Agilent Technologies) according to the protocol of the Agilent Seahorse XF Glycolysis Stress Test Kit (14). Briefly, confluent cells were serum starved overnight, followed by administration of 100 nM insulin for 5 hours. Before analysis, cells were washed once with PBS, incubated in 175 μl XF base media (Agilent Technologies) supplemented with 2 mM glutamine with or without 100 nM insulin, and incubated at 37°C without CO_2_ for 1 hour. Extracellular acidification rate (ECAR) values were measured in the basal state, after injection of glucose, oligomycin and 2-deoxyglucose (2-DG). The quantitative value was normalized by the respective protein content. Brown preadipocytes were differentiated, and lipid accumulation was quantified by oil red O staining as described previously (49).

### Measurement of glucose uptake, cholesterol, and triglycerides.

Cells were FBS starved for 6 hours with DMEM containing 0.1% BSA and incubated with KRH buffer (4 mM KH_2_PO_4_, 1 mM MgSO_4_, 1 mM CaCl_2_, 120 mM NaCl, 10 mM NaHCO_3_, 30 mM HEPES, pH 7.4) for 20 minutes. Cells were then treated with insulin at final concentrations of 0, 1, 10, or 100 nM for 20 minutes. Glucose uptake was assessed using 2-NBDG (Thermo Fisher Scientific) at a final concentration of 100 μM for 1 hour. After incubation, cells were washed twice with PBS and the signal read using a fluorescence plate reader at excitation of approximately 465 nm and emission of approximately 540 nm. Plasma and liver cholesterol levels were measured using Total Cholesterol Assay Kits (STA-384, Cell Biolabs Inc.). Plasma and liver triglyceride levels were measured using the Triglyceride Colorimetric Assay Kit (10010303, Cayman).

### Receptor internalization assay.

Ligand-induced internalization of receptors was analyzed as described previously (14). Briefly, preadipocytes expressing WT-IR and L973F-IR were serum starved in high-glucose DMEM supplemented with 0.1% BSA for 3 hours, followed by stimulation with 100 nM insulin for 0, 30, and 120 minutes. The cells were rinsed once with ice-cold PBS, followed by 0.5 mg/ml EZ-Link Sulfo-NHS-LC-Biotin (Thermo Fisher Scientific) labeling at 4°C for 30 minutes according to the manufacturer’s protocol. The cells were lysed in RIPA buffer (MilliporeSigma) supplemented with protease and phosphatase inhibitors (BioTools). Biotinylated surface proteins were enriched by incubating 120 μg total protein lysates with 15 μl Pierce Streptavidin Agarose Resins (Thermo Fisher Scientific) in 700 ml total volume on a rotator at 4°C for 1 hour. The beads were then washed with RIPA lysis buffer 3 times, and bound proteins were eluted by boiling in SDS-PAGE loading buffer. Cell-surface receptors were detected by immunoblotting using the IRβ antibody (catalog sc-711).

### Phosphoproteome analysis.

For phosphoproteomics, incubated WT-IR and L973F-IR preadipocytes were processed as described previously (15, 26, 50). These cell lysates in SDC buffer (4% SDC in 100 mM Tris-HCl pH8.5) were also used for immunoblotting. The enrichment of phosphopeptides as measured by liquid chromatography–tandem mass spectrometry (LC-MS/MS) and the output phospho table was processed as described previously (15, 50). After filtering rows that had more than 50% missing values, we normalized the data to make the samples have the same median log intensity. Then, the remaining missing values were imputed using a random forest algorithm in the R package missForest. To discover the differentially regulated phosphosites, the statistical significance of phosphopeptides was assessed with limma, an R package that powers differential expression analyses (51). A moderated *F* test was performed to detect phosphosites that were differentially expressed among 6 groups. For hierarchical clustering, the top phosphosites that had an FDR of less than 0.05 using the *F* tests were selected. Then hierarchical cluster analysis was performed based on the Euclidean distance of these selected phosphosites. Pathway enrichment analyses of phosphosites were done using STRING (https://string-db.org). The MS proteomics raw data and the corresponding processing reports generated in this study have been deposited in the ProteomeXchange Consortium database (http://proteomecentral.proteomexchange.org) via the PRIDE (52) partner repository (PXD038649).

### Statistics.

Comparisons between more than 2 groups were performed using 1-way ANOVA followed by Tukey’s multiple-comparison test or Dunnett’s multiple-comparison test. Differences between the 2 groups were determined using 2-tailed Student’s *t* test. Comparisons between 2 groups and 2 nominal variables were made using 2-way ANOVA followed by Šidák’s multiple-comparison test. In all cases, differences were considered significant when the *P* value was less than 0.05.

### Study approval.

All animal study protocols described in this study were approved by the Institutional Animal Care and Use Committee at the Joslin Diabetes Center (Harvard Medical School, Boston, MA).

## Author contributions

HN designed research, performed experiments, analyzed the data, and wrote the manuscript. WC, BBB, and AKG helped with experiments and reviewed and edited the manuscript. FTW generated the mouse model. NJWA and MS performed phosphoproteomic analysis. HP and JMD performed bioinformatics analysis. MM supervised phosphoproteome analysis. CRK designed research, wrote the manuscript, and supervised the project.

## Supplementary Material

Supplemental data

Supplemental table 1

## Version 1 12/22/2022

In-Press Preview

## Version 2 02/15/2023

Electronic publication

## References

[1] Boucher J (2014). Insulin receptor signaling in normal and insulin-resistant states. Cold Spring Harb Perspect Biol.

[2] Kahn CR (1974). Quantitative aspects of the insulin-receptor interaction in liver plasma membranes. J Biol Chem.

[3] Steele-Perkins G (1988). Expression and characterization of a functional human insulin-like growth factor I receptor. J Biol Chem.

[4] Gutmann T (2018). Visualization of ligand-induced transmembrane signaling in the full-length human insulin receptor. J Cell Biol.

[5] White MF, Kahn CR (2021). Insulin action at a molecular level — 100 years of progress. Mol Metab.

[6] Xu Y (2018). How ligand binds to the type 1 insulin-like growth factor receptor. Nat Commun.

[7] Saltiel AR (2021). Insulin signaling in health and disease. J Clin Invest.

[8] Hosoe J (2017). Structural basis and genotype-phenotype correlations of INSR mutations causing severe insulin resistance. Diabetes.

[9] Kadowaki T (1990). Five mutant alleles of the insulin receptor gene in patients with genetic forms of insulin resistance. J Clin Invest.

[10] Accili D (1996). Early neonatal death in mice homozygous for a null allele of the insulin receptor gene. Nat Genet.

[11] Liu JP (1993). Mice carrying null mutations of the genes encoding insulin-like growth factor I (Igf-1) and type 1 IGF receptor (Igf1r). Cell.

[12] Abuzzahab MJ (2003). IGF-I receptor mutations resulting in intrauterine and postnatal growth retardation. N Engl J Med.

[13] Juanes M (2015). Three novel IGF1R mutations in microcephalic patients with prenatal and postnatal growth impairment. Clin Endocrinol (Oxf).

[14] Cai W (2017). Domain-dependent effects of insulin and IGF-1 receptors on signalling and gene expression. Nat Commun.

[15] Nagao H (2021). Distinct signaling by insulin and IGF-1 receptors and their extra- and intracellular domains. Proc Natl Acad Sci U S A.

[16] Gustafson TA (1995). Phosphotyrosine-dependent interaction of SHC and insulin receptor substrate 1 with the NPEY motif of the insulin receptor via a novel non-SH2 domain. Mol Cell Biol.

[17] White MF (1988). Mutation of the insulin receptor at tyrosine 960 inhibits signal transmission but does not affect its tyrosine kinase activity. Cell.

[18] Longo N (2002). Genotype-phenotype correlation in inherited severe insulin resistance. Hum Mol Genet.

[19] Stolt PC, Bock HH (2006). Modulation of lipoprotein receptor functions by intracellular adaptor proteins. Cell Signal.

[20] Backer JM (1992). The insulin receptor juxtamembrane region contains two independent tyrosine/beta-turn internalization signals. J Cell Biol.

[21] Chen WJ (1990). NPXY, a sequence often found in cytoplasmic tails, is required for coated pit-mediated internalization of the low density lipoprotein receptor. J Biol Chem.

[22] Prager D (1994). Human insulin-like growth factor I receptor internalization. Role of the juxtamembrane domain. J Biol Chem.

[23] Chen Y (2019). Insulin receptor trafficking: consequences for insulin sensitivity and diabetes. Int J Mol Sci.

[24] Trischitta V (1989). Defects in insulin-receptor internalization and processing in monocytes of obese subjects and obese NIDDM patients. Diabetes.

[25] Wiley HS, Burke PM (2001). Regulation of receptor tyrosine kinase signaling by endocytic trafficking. Traffic.

[26] Humphrey SJ (2018). High-throughput and high-sensitivity phosphoproteomics with the EasyPhos platform. Nat Protoc.

[27] Stepniak E (2006). c-Jun/AP-1 controls liver regeneration by repressing p53/p21 and p38 MAPK activity. Genes Dev.

[28] Hammouda MB (2020). The JNK signaling pathway in inflammatory skin disorders and cancer. Cells.

[29] Malanga D (2015). The Akt1/IL-6/STAT3 pathway regulates growth of lung tumor initiating cells. Oncotarget.

[30] Cai W (2021). Peripheral insulin regulates a broad network of gene expression in hypothalamus, hippocampus, and nucleus accumbens. Diabetes.

[31] Czech MP (2017). Insulin action and resistance in obesity and type 2 diabetes. Nat Med.

[32] Khan S (2021). Targeting hypercoagulation to alleviate Alzheimer’s disease progression in metabolic syndrome. Int J Obes (Lond).

[33] Lambie M (2021). Insulin resistance in cardiovascular disease, uremia, and peritoneal dialysis. Trends Endocrinol Metab.

[34] Steculorum SM (2014). The paradox of neuronal insulin action and resistance in the development of aging-associated diseases. Alzheimers Dement.

[35] David A (2011). Evidence for a continuum of genetic, phenotypic, and biochemical abnormalities in children with growth hormone insensitivity. Endocr Rev.

[36] Klammt J (2011). IGF1R mutations as cause of SGA. Best Pract Res Clin Endocrinol Metab.

[37] Cox OT (2015). IGF-1 receptor and adhesion signaling: an important axis in determining cancer cell phenotype and therapy resistance. Front Endocrinol (lausanne).

[38] He W (1995). Distinct modes of interaction of SHC and insulin receptor substrate-1 with the insulin receptor NPEY region via non-SH2 domains. J Biol Chem.

[39] Boucher J (2014). Insulin and insulin-like growth factor 1 receptors are required for normal expression of imprinted genes. Proc Natl Acad Sci U S A.

[40] Boucher J (2010). A kinase-independent role for unoccupied insulin and IGF-1 receptors in the control of apoptosis. Sci Signal.

[41] Liao L (2006). Liver-specific overexpression of the insulin-like growth factor-I enhances somatic growth and partially prevents the effects of growth hormone deficiency. Endocrinology.

[42] McMullen JR (2004). The insulin-like growth factor 1 receptor induces physiological heart growth via the phosphoinositide 3-kinase(p110alpha) pathway. J Biol Chem.

[43] Musaro A (2001). Localized Igf-1 transgene expression sustains hypertrophy and regeneration in senescent skeletal muscle. Nat Genet.

[44] Reiss K (1996). Overexpression of insulin-like growth factor-1 in the heart is coupled with myocyte proliferation in transgenic mice. Proc Natl Acad Sci U S A.

[45] Zhu B (2001). Targeted overexpression of IGF-I in smooth muscle cells of transgenic mice enhances neointimal formation through increased proliferation and cell migration after intraarterial injury. Endocrinology.

[46] Boucher J (2016). Differential roles of insulin and IGF-1 receptors in adipose tissue development and function. Diabetes.

[47] O’Neill BT (2015). Differential role of insulin/IGF-1 receptor signaling in muscle growth and glucose homeostasis. Cell Rep.

[48] O’Neill BT (2016). Insulin and IGF-1 receptors regulate FoxO-mediated signaling in muscle proteostasis. J Clin Invest.

[49] Boucher J (2010). Insulin and insulin-like growth factor-1 receptors act as ligand-specific amplitude modulators of a common pathway regulating gene transcription. J Biol Chem.

[50] Batista TM (2020). A cell-autonomous signature of dysregulated protein phosphorylation underlies muscle insulin resistance in type 2 diabetes. Cell Metab.

[51] Ritchie ME (2015). Limma powers differential expression analyses for RNA-sequencing and microarray studies. Nucleic Acids Res.

[52] Vizcaino JA (2016). 2016 update of the PRIDE database and its related tools. Nucleic Acids Res.

