# Supplementary material for: Leucine-973 is a crucial residue differentiating insulin and IGF-1 receptor signaling
Source: J Clin Invest. 2023 Feb 15;133(4):e161472. doi: 10.1172/JCI161472 (PMC9927934; doi:10.1172/JCI161472)
Supplement: Supplemental data [file jci-133-161472-s027.pdf]

Supplemental Figure 1

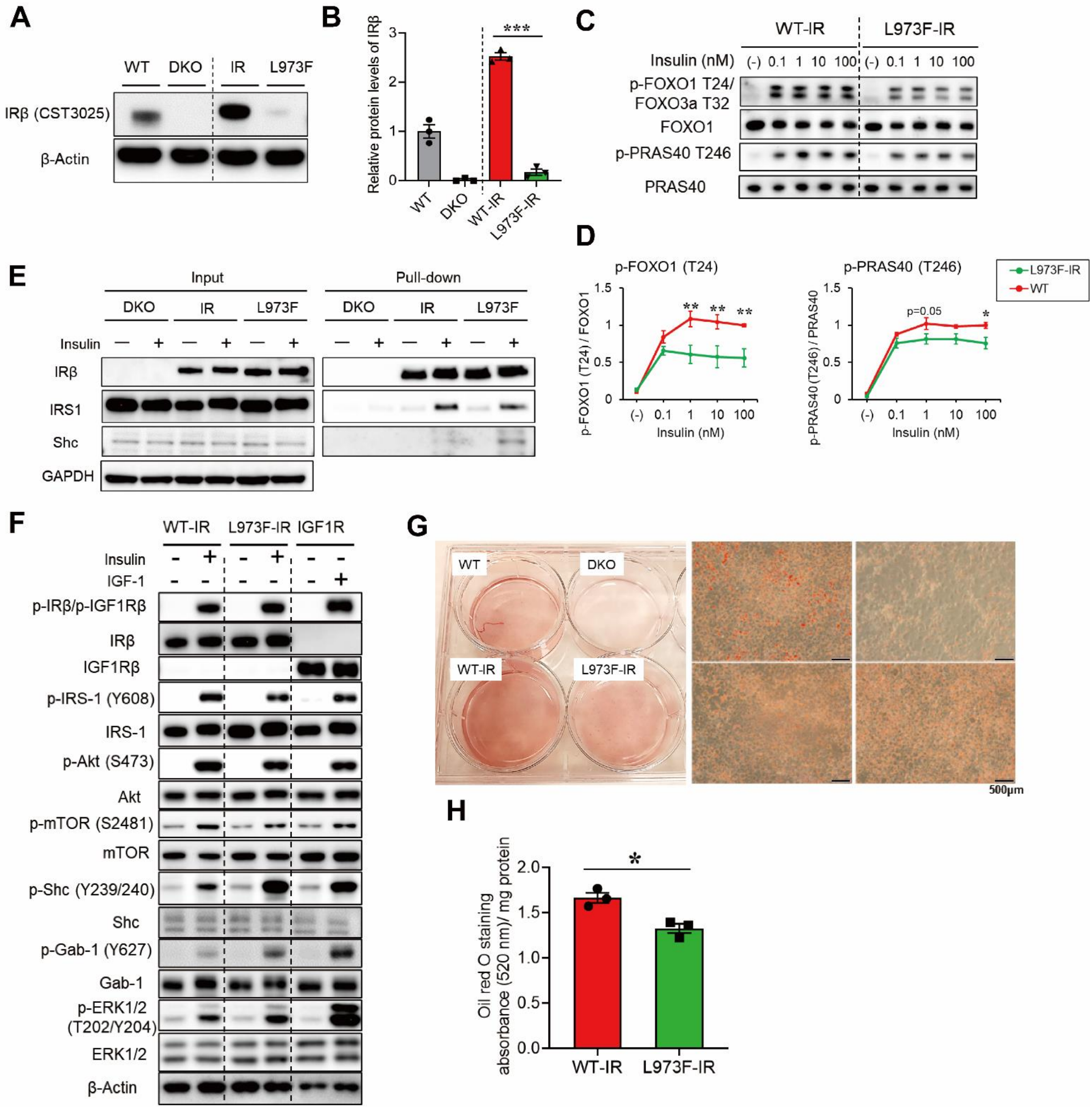

Supplemental Figure 1. Differential roles between WT-IR and L973F-IR.
(A) Immunoblotting of IR using an antibody specific for IRβ (CST3025, Cell Signaling Technology) lysates from WT, DKO, WT-IR and L973F-IR cells. CST3025 corresponds to residues surrounding tyrosine^{972} of IRβ. (B) Densitometric analysis of IRβ (CST3025, Cell Signaling Technology) by immunoblotting. The level of IRβ in WT cells was set at 1. Data are means ± SEM (n=3 per group). (C) Immunoblotting of the phosphorylation of Foxo1 and PRAS40 in lysates from preadipocytes expressing WT-IR and L973F-IR stimulated with 0, 0.1, 1, 10 or 100nM insulin for 15 min. (D) Densitometric analysis of phosphorylated Foxo1 and PRAS40 following insulin stimulation. The level of each phosphorylated protein in WT-IR cells stimulated with 100 nM insulin was set at 1. Data are means ± SEM (n=4 per group). * $P < 0.05$, ** $P < 0.01$ WT-IR vs L973F-IR, two-way ANOVA. (E) Flag-tagged receptor-containing protein complexes were pull-down with anti-Flag M2 magnetic beads (M8823, Millipore) following 100 nM insulin stimulation for 15 min. Bound proteins were detected by immunoblotting. (F) Immunoblotting of the phosphorylation of IRβ/IGF1Rβ, IRS-1, Akt, mTOR, Shc, Gab-1 and ERK in lysates from preadipocytes expressing WT-IR, L973F-IR and IGF1R. WT-IR and L973F-IR cells were stimulated with 100 nM insulin for 15 min, while IGF1R cells were stimulated with 100nM IGF-1 for 15 min. (G) Oil red O staining images of WT, DKO, WT-IR and L973F-IR cells day 7 after induction of differentiation. Scale bars; 500 µm. (H) Triglyceride accumulation in WT-IR and L973F-IR cells normalized by protein content. Oil red O staining day 7 after induction of differentiation (n=3 per group). Data are means ± SEM. * $P < 0.05$, unpaired Student's *t* test.

Supplemental Figure 2

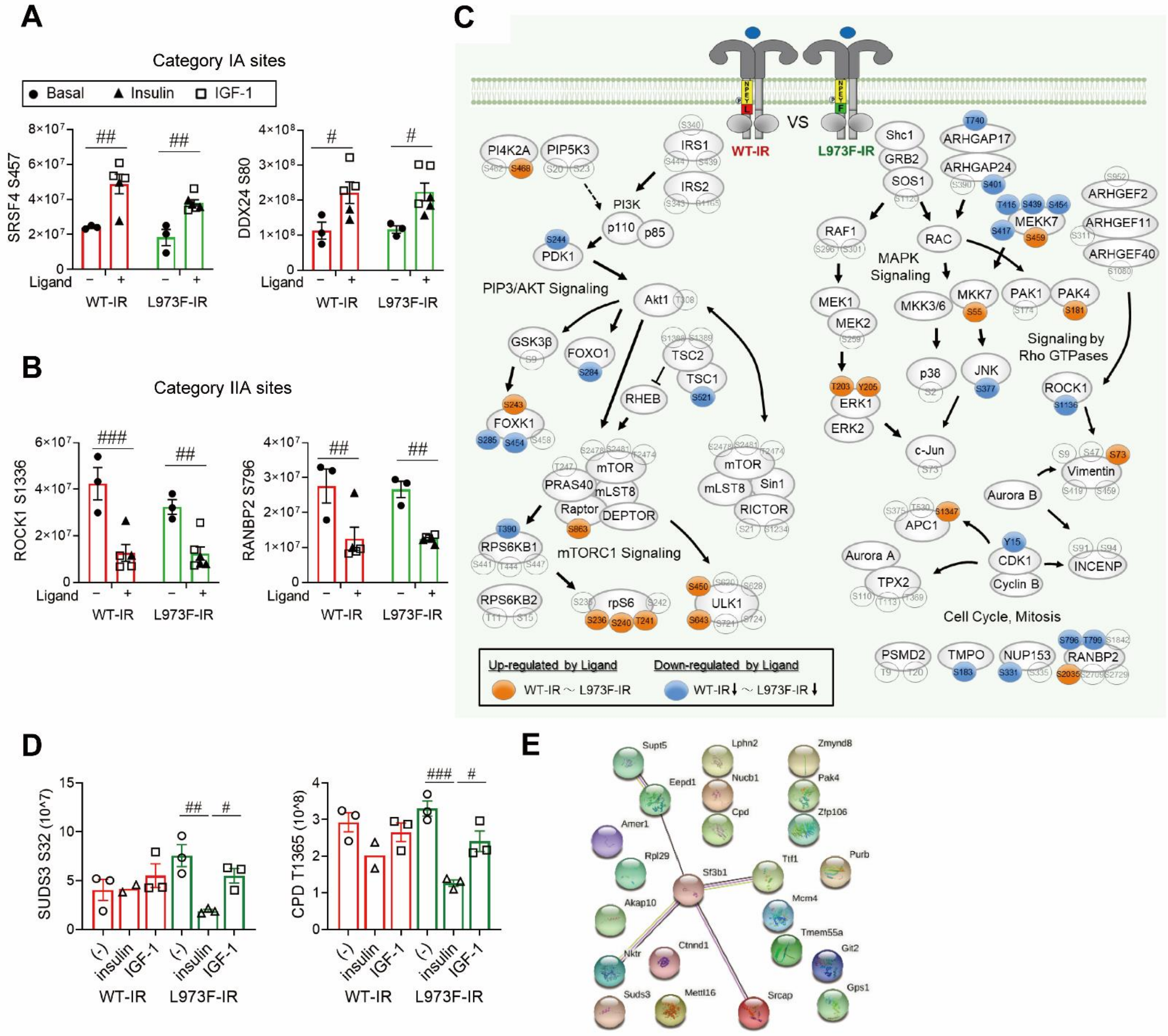

Supplemental Figure 2.Phosphoproteomic signature of WT-IR and L973F-IR cells. Overlapping signaling events between WT-IR and L973F-IR.

(A) Quantification of exemplary phosphosites of equally upregulated by ligand stimulation for WT-IR and L973F-IR (Category I-A). Data are means ± SEM of phosphosite intensity values. # $P < 0.05$, ## $P < 0.01$ basal vs ligand, two-way ANOVA. (B) Quantification of exemplary phosphosites of equally downregulated by ligand stimulation for WT-IR and L973F-IR (Category II-A). Data are means ± SEM of phosphosite intensity values. ## $P < 0.01$, ### $P < 0.001$ basal vs ligand, two-way ANOVA. (C) Signaling map showing phosphosites equally up- or down-regulated by ligand in cells expressing WT-IR and L973F-IR as identified by phosphoproteomics. Orange-filled circles represent sites for which phosphorylation was increased, and blue-filled circles represent sites for which phosphorylation was decreased by ligand ($P < 0.05$). Arrows indicate known protein-protein interactions and phosphorylation/dephosphorylation events from the PhosphositePlus database and the literature. (D) Exemplary phosphosites those were downregulated more by insulin than IGF-1 in L973F-IR cells (Category II-D in Figure 3B). Data are means ± SEM of phosphosites intensity values. # $P < 0.05$, ### $P < 0.01$, ### $P < 0.001$, two-way ANOVA. (E) The protein interaction network of the Category II-D sites was obtained from the STRING database.

Supplemental Figure 3

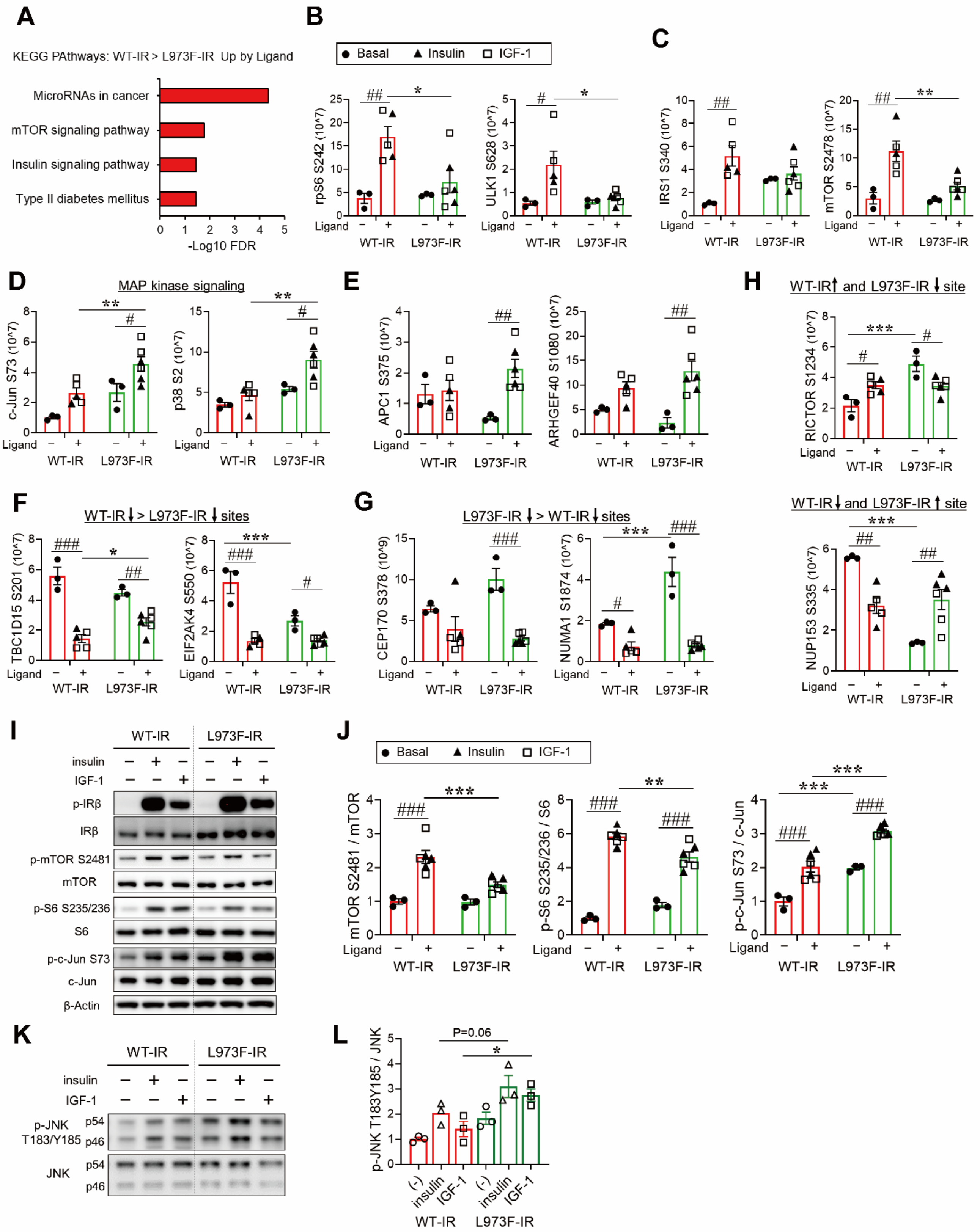

Supplemental Figure 3. WT-IR and L973F-IR specific signaling in the phosphoproteome. (A) KEGG pathway enrichment analysis of phosphosites up-regulated by ligand for fold-change WT-IR > L973F-IR. Plots are –log10 transforms of enrichment FDR value. (B) Quantification of exemplary phosphosites in mTOR signaling pathway. Data are means ± SEM of phosphosites intensity values. # $P < 0.05$, ## $P < 0.01$ basal vs ligand, * $P < 0.05$ WT-IR vs L973F-IR, two-way ANOVA. (C) Quantification of some important phosphosites up-regulated by ligand (for this analysis we have combined the insulin and IGF-1 stimulated samples since they showed similar changes) for fold-change WT-IR > L973F-IR corresponding to Fig. 4A. (D) Quantification of exemplary phosphosites in MAP kinase signaling pathway. Data are means ± SEM of phosphosites intensity values. # $P < 0.05$ basal vs ligand, * $P < 0.05$, ** $P < 0.01$ WT-IR vs L973F-IR, two-way ANOVA. (E) Quantification of exemplary phosphosites up-regulated by ligand showing fold-change L973F-IR > WT-IR and corresponding to Fig. 4B. (F) Quantification of example phosphosites down-regulated by ligand for group with fold-change L973-IR > WT-IR and corresponding to Fig. 4C. (G) Quantification of example phosphosites down-regulated by ligand for group with fold-change WT > L973F-IR corresponded to Fig. 4D. (H) Quantification of exemplary phosphosites showing opposite regulation by ligand between WT-IR and L973F-IR and corresponding to Fig. 4E. Data in (B-H) are means ± SEM of phosphosites intensity values. # $P < 0.05$, ## $P < 0.01$, ### $P < 0.001$ basal vs ligand, * $P < 0.05$, ** $P < 0.01$, *** $P < 0.001$ WT-IR vs L973F-IR, two-way ANOVA. (I and J) Validation of phosphoproteomics by immunoblotting in lysates from WT-IR and L973F-IR cells for phospho-mTOR^{S2481}, phospho-S6$^{S235/236}$ and phospho-c-Jun^{S73} (I), and quantification of phosphorylation of mTOR^{S2481}, S6$^{S235/236}$ and c-Jun^{S73} (J). The level of each phosphorylated protein in WT-IR cells in the basal state was set at 1. Data are means ± SEM (n=3 per group). ### $P < 0.001$ basal vs ligand, ** $P < 0.01$, *** $P < 0.001$ two-way ANOVA. (K and L) Phosphorylation of JNK by immunoblotting in lysates from WT-IR and L973F-IR cells. Representative images of immunoblot analysis of p-JNK$^{T183/Y185}$ and JNK (K) and quantification of p-JNK$^{T183/Y185}$ (L). The level in WT-IR cells in the basal state was set at 1.

Supplemental Figure 4

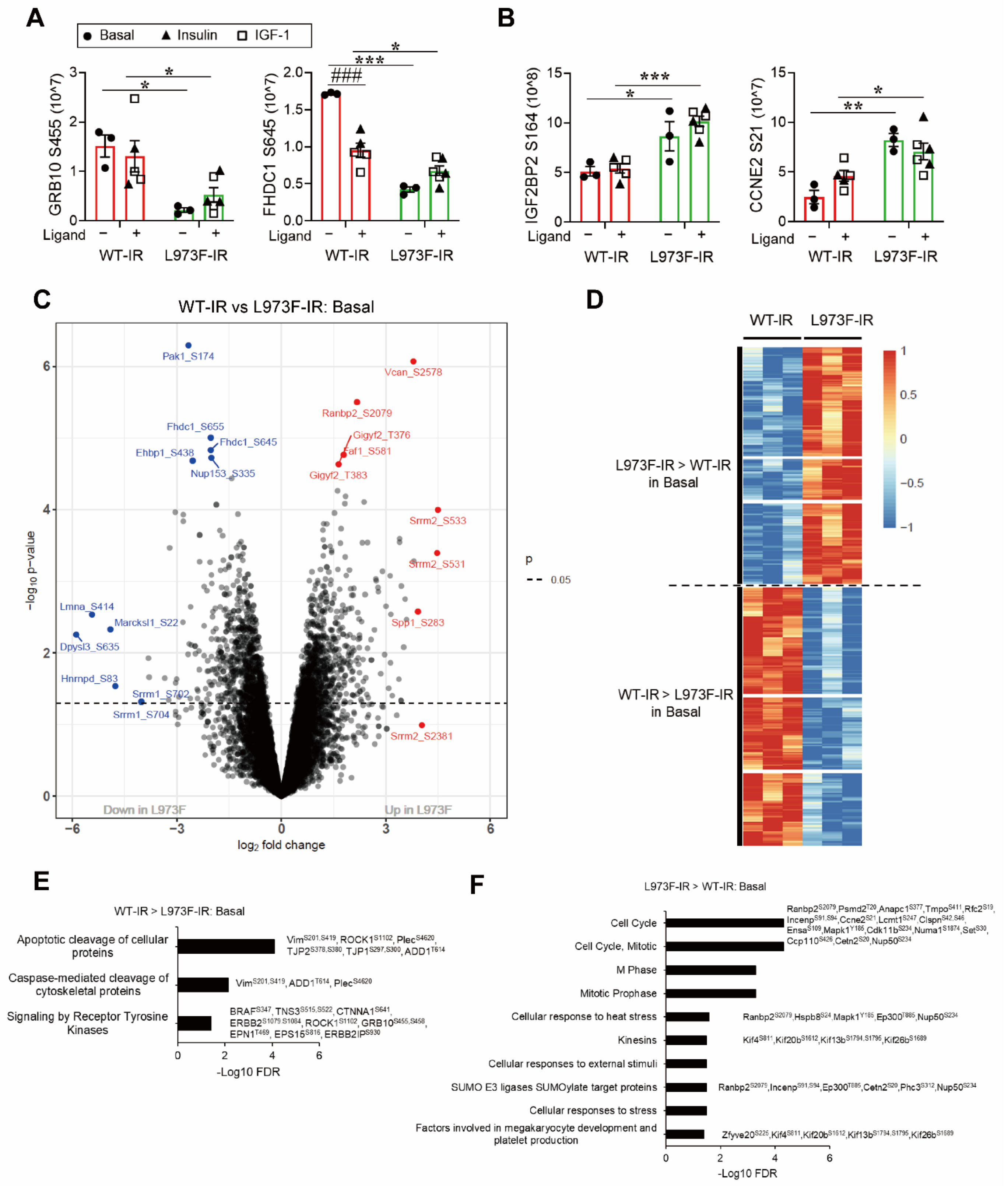

Supplemental Figure 4. Differential phosphoproteomic patterns by WT-IR and L973F-IR in the basal state.

(A) Quantification of exemplary phosphosites those were not regulated by ligand but significantly higher at the basal in WT-IR cells compared with L973F-IR cells. (B) Quantification of exemplary phosphosites those were not regulated by ligand but significantly higher at the basal in L973F-IR cells compared with WT-IR cells. Data in (A and B) are means ± SEM. ### $P < 0.001$ basal vs ligand, * $P < 0.05$, ** $P < 0.01$, *** $P < 0.001$ WT-IR vs L973F-IR, two-way ANOVA. (C) Volcano plot of phosphoproteomic data on WT-IR and L973F-IR cells in the basal (non-stimulated by ligand) state. (D) Heatmap showing the hierarchical clustering of the phosphopeptides in WT-IR and L973F-IR expressing cells in the basal state (FDR < 0.25). Values are Z-scores of log2 transformed intensity values. (E) REACTOME pathway enrichment analysis of phosphosites in the WT-IR > L973F-IR: Basal cluster. Plots are –log10 transforms of enrichment FDR value. Included phosphosites of proteins in each pathway are also shown. (F) REACTOME pathway enrichment analysis of phosphosites in the L973F-IR > WT-IR: Basal cluster. Plots are –log10 transforms of enrichment FDR value. Included phosphosites of proteins in some important pathways are also shown.

Supplemental Figure 5

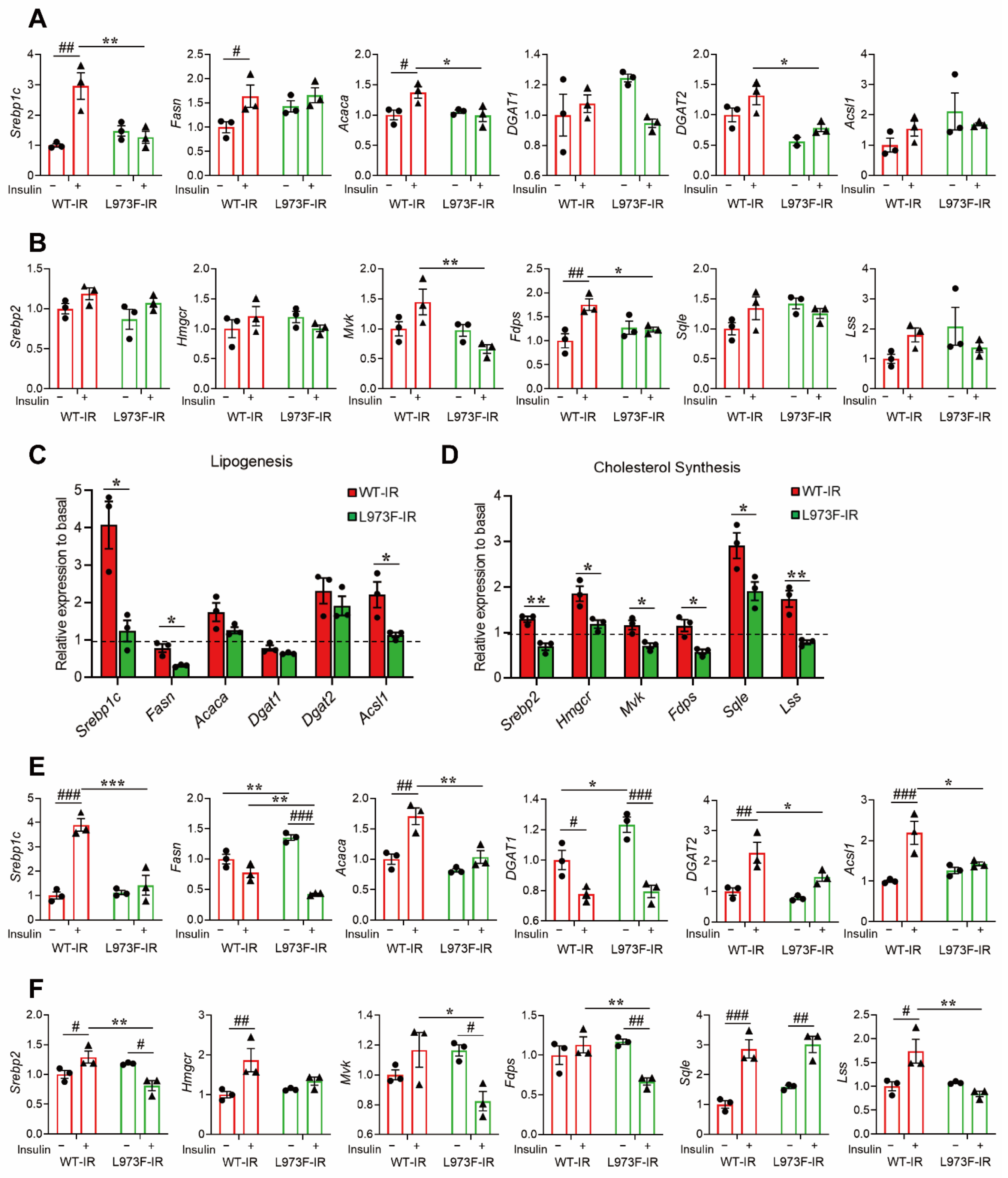

Supplemental Figure 5. Effects of the L973F substitution in the IR on gene transcription related fatty acid synthesis and cholesterol synthesis.

(A and B) Relative mRNA expression of genes associated with fatty acid synthesis (A) and cholesterol biosynthesis (B) in WT-IR and L973F-IR preadipocytes. The fold changes of expression in response to insulin stimulation are shown in Fig. 5E and F. WT-IR and L973F-IR preadipocytes were stimulated with or without 100 nM insulin for 6 h after 6 h FBS starvation. Gene expression levels of WT-IR preadipocytes at the basal were set at 1. (C and D) Fold change of expression in response to insulin stimulation for genes associated with fatty acid synthesis (C) and cholesterol biosynthesis (D) in WT-IR and L973F-IR differentiated adipocytes. WT-IR and L973F-IR adipocytes were stimulated with or without 100 nM insulin for 6 h following 6 h FBS starvation. Data are shown as means ± SEM. (E and F) Relative mRNA expression of genes associated with fatty acid synthesis (E) and cholesterol biosynthesis (F) in WT-IR and L973F-IR differentiated adipocytes. These fold changes of expression in response to insulin stimulation are shown in Fig. S5, C and D. Gene expression levels of WT-IR differentiated adipocytes at the basal were set at 1. # $P < 0.05$, ## $P < 0.01$, ### $P < 0.001$ basal vs ligand, * $P < 0.05$, ** $P < 0.01$ WT-IR vs L973F-IR, Student's t test for fold change analysis and two-way ANOVA for others.

Supplemental Figure 6

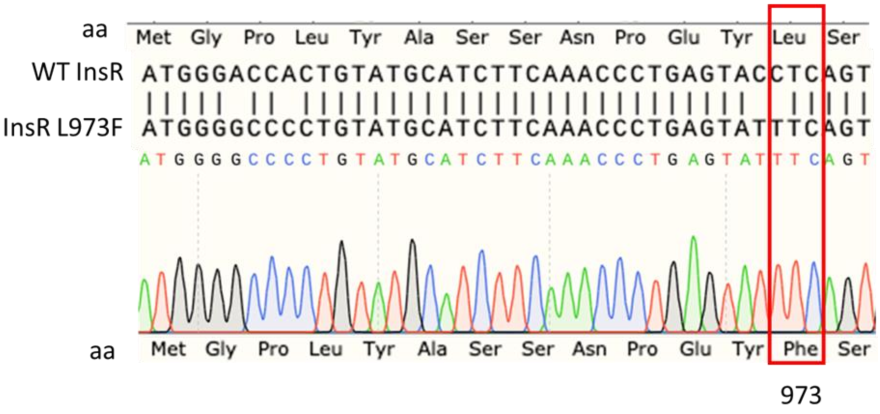

Supplemental Figure 6. Generation of a knockin mouse with the *INSR* L973F.
The DNA sequence chromatogram of genomic DNA isolated from L973F-IR mouse confirming the mutation.

Supplemental Figure 7

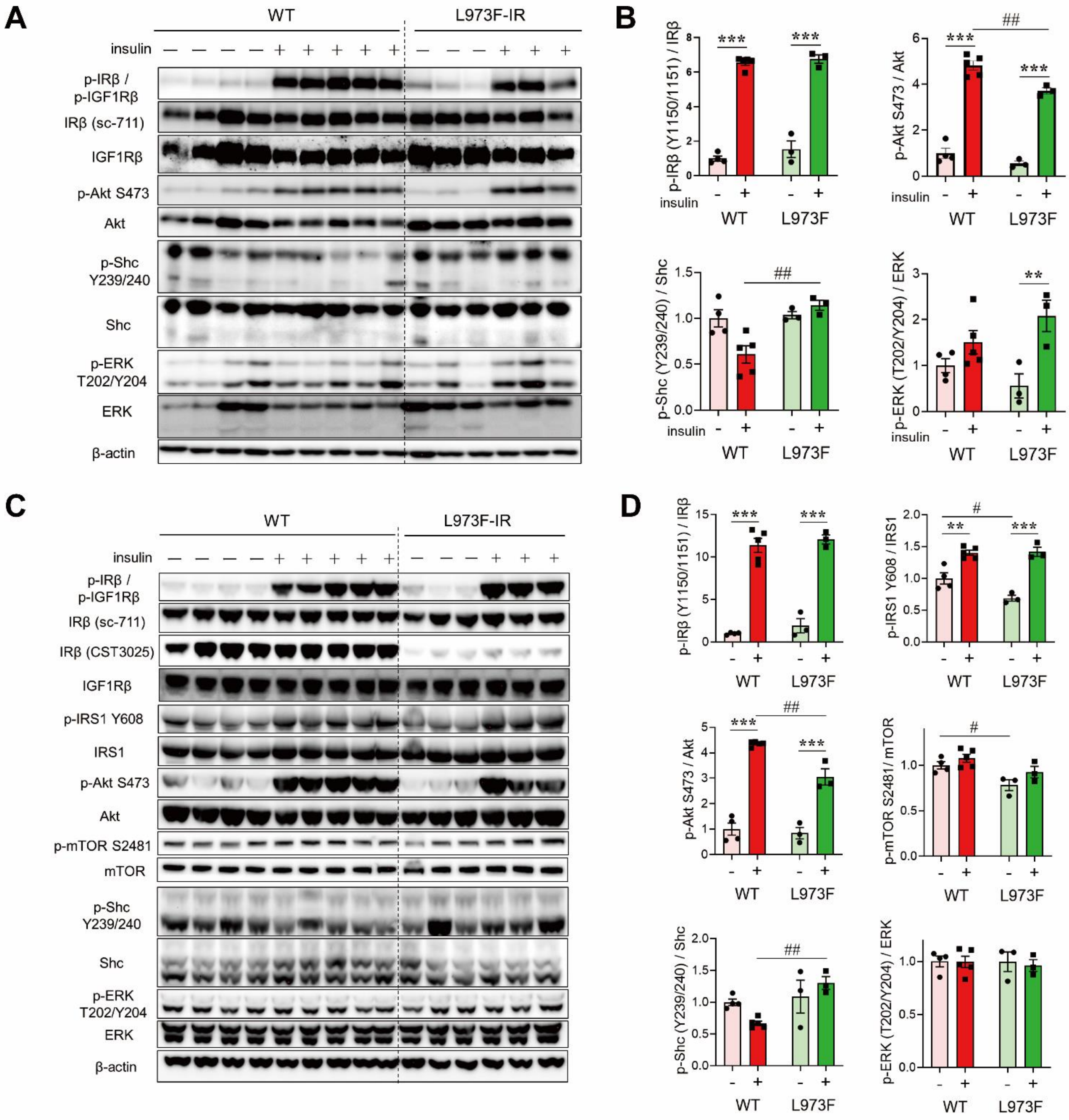

Supplemental Figure 7. Effects of the L973F substitution on insulin receptor signaling in mice.

(A) Analysis of insulin signaling in epididymal WAT of WT and L973F-IR chow-fed mice (7 months old male) administered insulin (2 U) or saline for 10 min. Immunoblot analysis was performed with the indicated antibodies. (B) Quantification of p-IRβ, p-Akt^{S473}, p-Shc$^{Y239/240}$ and p-ERK$^{T202/Y204}$. Results are presented as mean ± SEM. ** P < 0.01, *** P < 0.001 basal vs insulin, ### P < 0.001 WT vs L973F-IR mice, two-way ANOVA. (C) Analysis of insulin signaling in the liver of WT and L973F-IR chow-fed mice (7 months old male) administered insulin (2 U) or saline for 10 min. Immunoblot analysis was performed with the indicated antibodies. The images for IRβ and β-actin are same as in Figure 6C. (D) Quantification of p-IRβ, p-IRS1^{Y608}, p-Akt^{S473}, p-mTOR^{S2481}, p-Shc$^{Y239/240}$ and p-ERK$^{T202/Y204}$. Results are presented as mean ± SEM. ** P < 0.01, *** P < 0.001 basal vs insulin, ## P < 0.01, ### P < 0.001 WT vs L973F-IR mice, two-way ANOVA.

Supplemental Figure 8

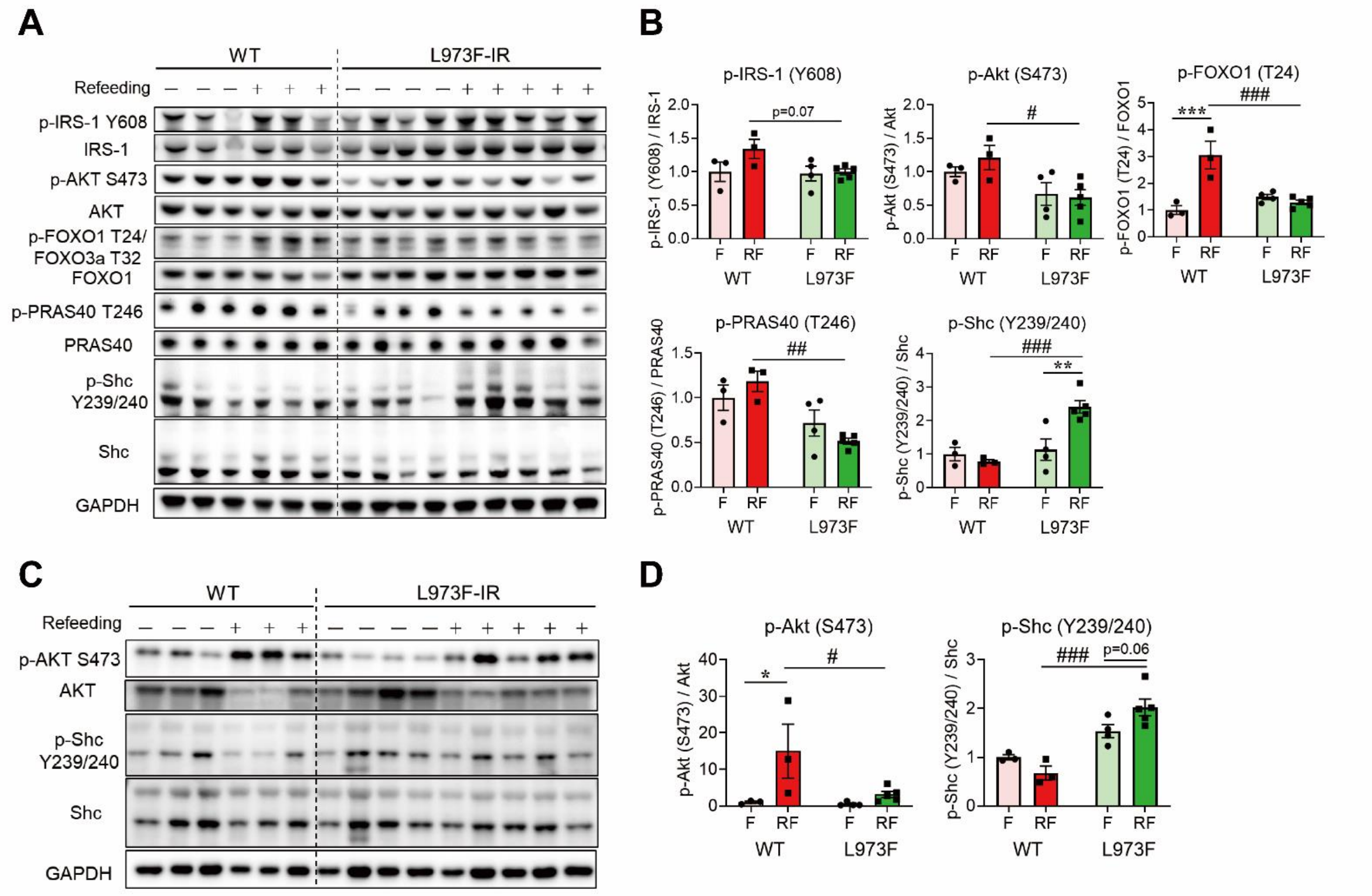

Supplemental Figure 8. Effects of the L973F substitution on insulin receptor signaling in mice during fasting and refeeding.

(A) Immunoblot analysis of insulin signaling in liver of WT and L973F-IR mice (6 months old male) starved overnight (15 h) and then refed for 2 h followed by sacrifice and tissue harvest. F; fasting (not refed) mice, RF; refed mice. (B) Quantification of p-IRS1^{Y608}, p-Akt^{S473}, p-Foxo1^{T24}, p-PRAS40^{T246} and p-Shc$^{Y239/240}$ in Fig. S8A. The level of each phosphorylated protein in WT fasting mice was set at 1. Results are presented as mean ± SEM. ** $P < 0.01$, *** $P < 0.001$ F vs RF, # $P < 0.05$, ## $P < 0.01$, ### $P < 0.001$ WT vs L973F-IR mice, two-way ANOVA. (C) Immunoblot analysis of insulin signaling in epididymal WAT of the fasting/refeeding WT and L973F-IR mice. (D) Quantification of p-Akt^{S473} and p-Shc$^{Y239/240}$ in Fig. S8C. The level of each phosphorylated protein in WT fasting mice was set at 1. Results are presented as mean ± SEM. * $P < 0.05$ F vs RF, # $P < 0.05$, ### $P < 0.001$ WT vs L973F-IR mice, two-way ANOVA.

Supplemental Figure 9

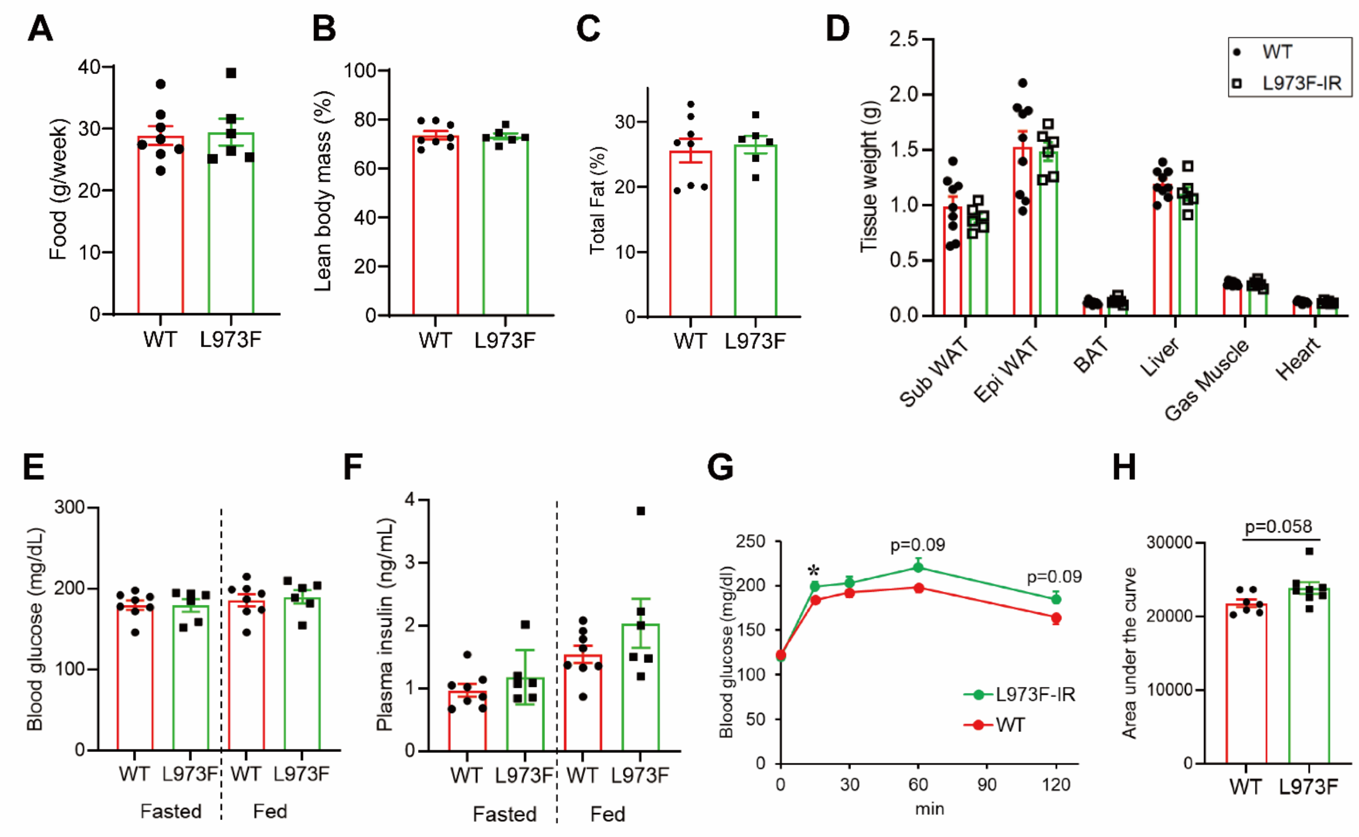

Supplemental Figure 9. The L973F mutation of IR in chow diet feeding male mice.
(A) Food intake (g/week) in control and L973F-IR chow-fed male mice (n=6-8). (B and C) Lean body mass (B) and total fat mass (C) in WT and L973F-IR male mice at age 5 months (n=6-8). (D) Tissue weight of 7-month-old chow-fed male mice (n=6-10). Sub WAT, subcutaneous WAT; Epi WAT, epididymal white adipose tissue; BAT, brown adipose tissue; Gas muscle, gastrocnemius muscle. (E) Blood glucose and (F) plasma insulin levels in the 6 h fasted and fed (ad libitum) states in chow-fed male mice (n=6-8). Data are means ± SEM. (G) Pyruvate tolerance tests in WT and L973F-IR chow-fed male mice fasted overnight. Blood glucose was monitored following intraperitoneal administration of 2 g/kg sodium pyruvate. Data are means ± SEM (n=7-8). * $P < 0.05$ WT vs L973F-IR mice, two-way ANOVA. (H) Area under the curve of blood glucose during pyruvate tolerance test. Data are means ± SEM. WT vs L973F-IR mice, unpaired Student's t test.

Supplemental Figure 10

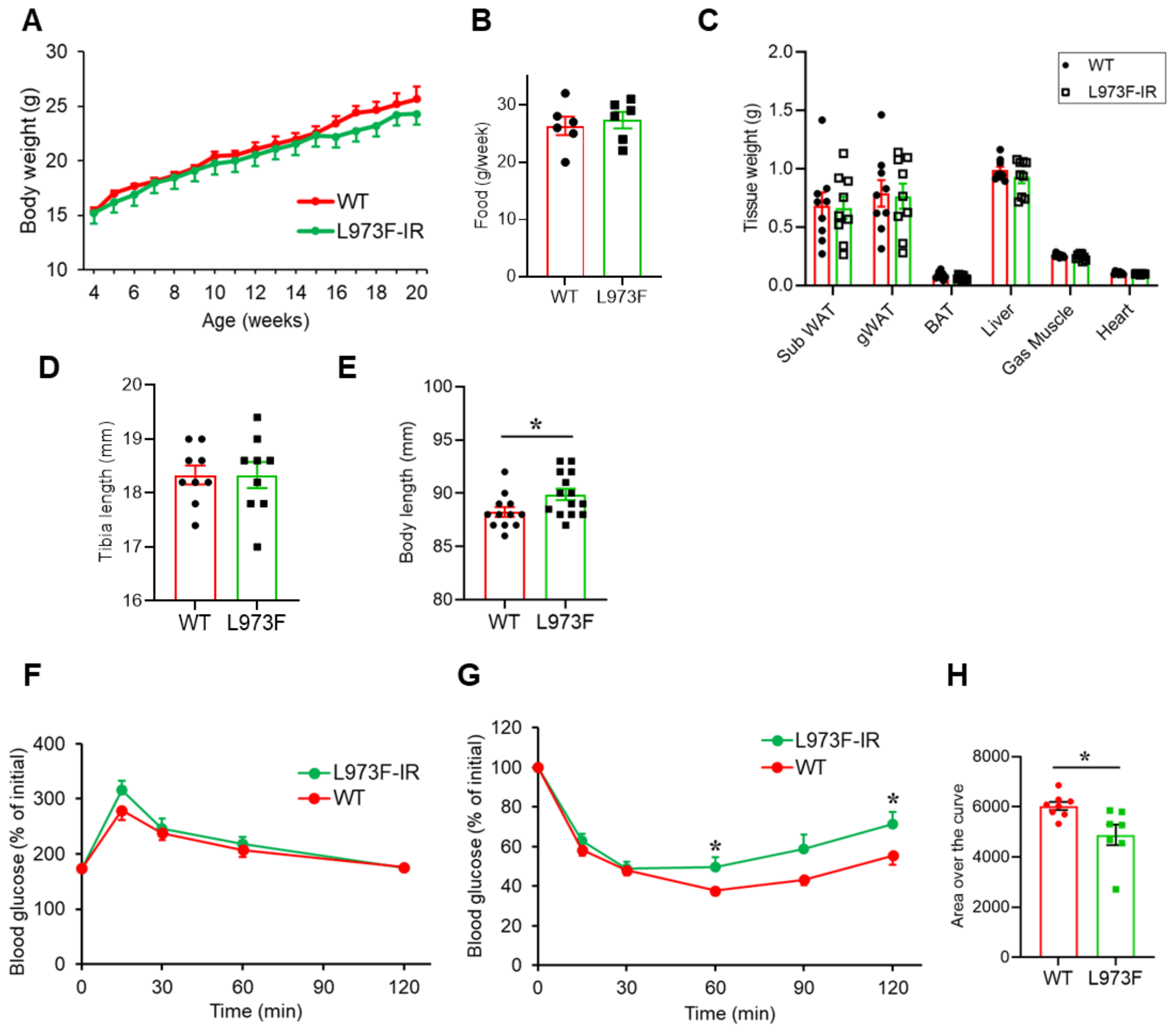

Supplemental Figure 10. The L973F mutation of IR in chow diet feeding female mice. (A) Body weight of WT and L973F-IR chow-fed female mice measured over the indicated time course. (B) Food intake (g/week) in WT and L973F-IR female mice (n=6). (C) Tissue weight of 5-month-old female mice (n=9). Sub WAT, subcutaneous WAT; gWAT, gonadal white adipose tissue; BAT, brown adipose tissue; Gas muscle, gastrocnemius muscle. (D) Tibia length as measured by DEXA scan of 9-week-old female mice (n=9). (E) Body length of 5 months old female animals (n=12-14). (F) Glucose tolerance test performed at 15 weeks of age in female animals (n=8). (G) Insulin tolerance test in female mice at 14 weeks of age (n=7-8). (H) Area over the curve of blood glucose during insulin tolerance test. Data in (A-H) are means ± SEM. * P < 0.05 WT vs L973F-IR mice, unpaired Student's *t* test.

Supplemental Figure 11

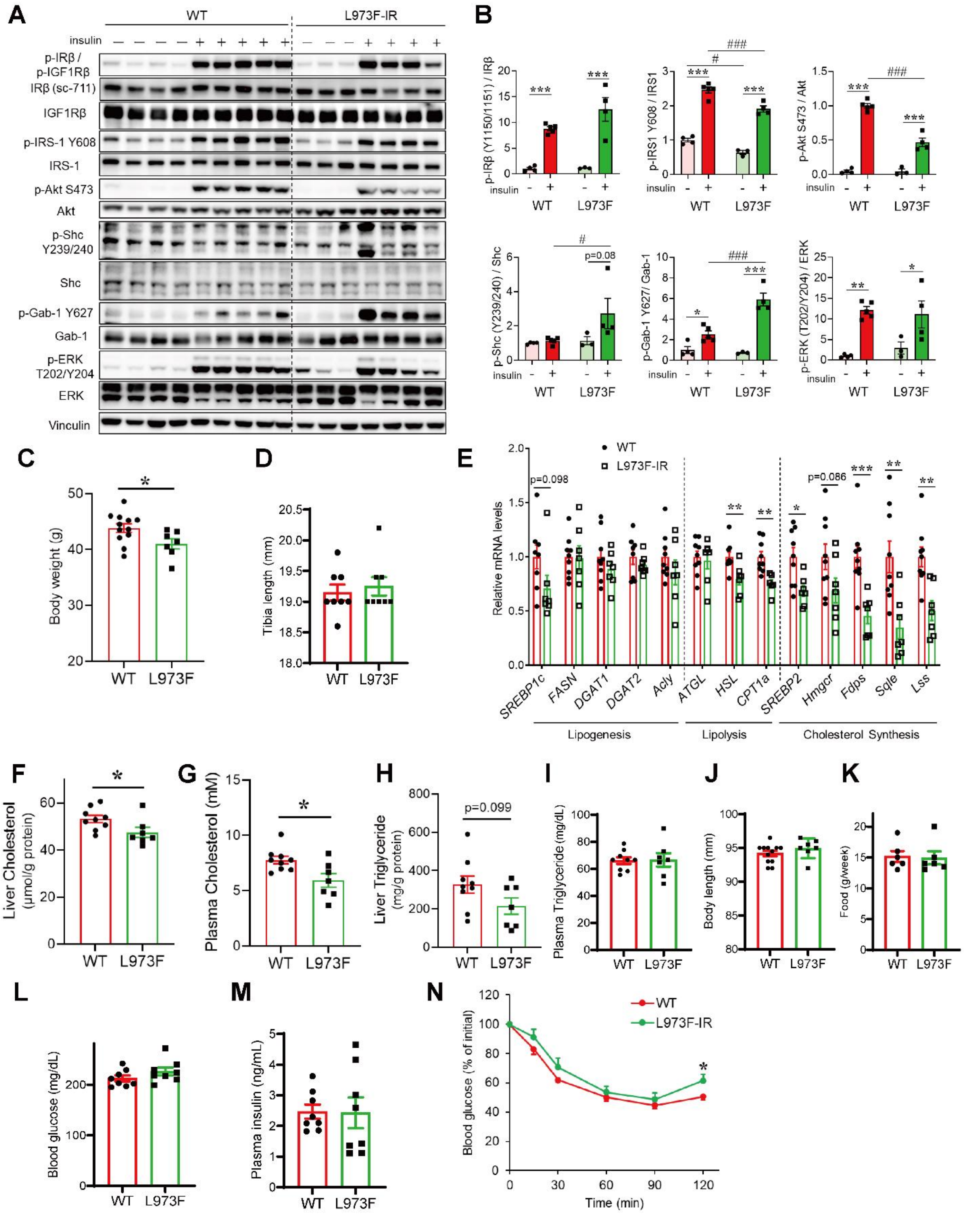

Supplemental Figure 11. High fat diet challenging in L973F-IR mutated male mice.

(A) Analysis of insulin signaling in skeletal muscle of WT and L973F-IR mice (5 months old male) administered insulin (2 U) or saline for 10 min. Mice were fed a high-fat diet (HFD, 60% of calories from fat) from 8 weeks of age to 20 weeks of age (3 months). Immunoblot analysis was performed with the indicated antibodies. (B) Quantification of p-IRβ, p-IRS1^{Y608}, p-Akt^{S473}, p-Shc$^{Y239/240}$, p-Gab-1^{Y627} and p-ERK$^{T202/Y204}$. Results are presented as mean ± SEM. * $P < 0.05$, ** $P < 0.01$, *** $P < 0.001$ basal vs insulin, # $P < 0.05$, ### $P < 0.001$ WT vs L973F-IR mice, two-way ANOVA. (C) Body weight at 21 weeks of age (n=7-12), and (D) food intake (g/week) at 16 weeks of age (n=6) in HFD fed male mice. (E) Changes in mRNA level of genes associated with lipogenesis, lipolysis and cholesterol synthesis in the liver of WT and L973F-IR HFD-fed male mice (n=7-9). Mice were fasted 6 h before collection of the liver. (F and G) Liver cholesterol (F) and plasma cholesterol levels (G) in HFD fed male mice (n=7-9). Mice were fasted 6 h before collection of plasma and the liver. (H and I) Liver triglyceride (H) and plasma triglyceride levels (fasted 6 h) (I) in HFD fed male mice (n=7-9). (J) Body length at 5 months of age (n=7-12), (K) Tibia length measured by DEXA scan at 17 weeks of age (n=8), (L) fasting (6 h) blood glucose (n=8), and (M) fasting plasma insulin (n=8) levels in HFD fed male mice. (N) Intraperitoneal insulin tolerance test in HFD-fed male mice at 16 weeks of age (n=7). Results are shown as mean ± SEM. * $P < 0.05$ WT vs L973F-IR mice, unpaired Student's *t* test.

Supplemental Table 1. Mouse primer sequences for PCR

Gene	Forward primer sequence (5'-3')	Reverse primer sequence (5'-3')
Insr	AAATGCAGGAACTCTCGGAAGCCT	ACCTTCGAGGATTTGGCAGACCTT
Igf1r	ATCGCGATTTCTGCGCCAACA	TTCTTCTCTTCATCGCCGCAGACT
Adiponectin	GGAGATGCAGGTCTTCTTGG	CGAATGGGTACATTGGGAAC
Pparg	TGTTATGGGTGAAACTCTGGG	AGAGCTGATTCCGAAGTTGG
Cebpa	CAAGAACAGCAACGAGTACCG	GTCACTGGTCAACTCCAGCAC
Srebp1c	GAGCCATGGATTGCACATTT	CTCAGGAGAGTTGGCACCTG
Fasn	CCCCTCTGTTAATTGGCTCC	TTGTGGAAGTGCAGGTTAGG
Dgat1	CCAACCATCTGATCTGGCTTA	CACTTGTGCACGGGGATATT
Dgat2	TCGCGAGTACCTGATGTCTG	CTTCAGGGTGACTGCGTTCT
Srebp2	GCAGCAACGGGACCATTCT	CCCCATGACTAAGTCCTTCAACT
Hmgcr	AGCTTGCCCGAATTGTATGTG	TCTGTTGTGAACCATGTGACTTC
Fdps	AGCCGAAGAAACAGGATGCTGAGA	TTCCAGAAGCAGAGCGTCGTTGAT
Sqle	TTGTTGCGGATGGACTCTTCTCCA	GTTGACCAGAACAAGCTCCGCAAA
Acaca	AAGGCTATGTGAAGGATGTGG	CTGTCTGAAGAGGTTAGGGAAG
Acsl1	AGAACTGTGCAGGAACAAGG	TCAGAAGGCCGTTGTCAATAG
Mvk	GGTGTGGTCGGAACTTCCC	CCTTGAGCGGGTTGGAGAC
Lss	TGTTCGTTGGTCAGTGGATG	GATAGCAAATGAAGTGTCCCAG
p53	GAAGTCCTTTGCCCTGAACTGCC	GTTTACGCCCGCGGATCTTG
p21	CACGTGGCCTTGTCGCTGTCTT	CGTGGGCACTTCAGGGTTTTCT
Ccl2	TTAAAAACCTGGATCGGAACCAA	GCATTAGCTTCAGATTTACGGGT
Il-6	GCTACCAAACTGGATATAATCAGGA	CCAGGTAGCTATGGTACTCCAGAA
Tbp	ACCCTTCACCAATGACTCCTATG	TGACTGCAGCAAATCGCTTGG
